# Exploring the Natural Products Atlas (NPAtlas) Database for Hunting Prospective Irreversible Covalent DprE1 Inhibitors With Antitubercular Activity: An Integrated *In-Silico* Approach

**DOI:** 10.1155/jotm/8879019

**Published:** 2026-02-14

**Authors:** Mahmoud A. A. Ibrahim, Doaa G. M. Mahmoud, Sherif S. Ebada, Peter A. Sidhom, Gamal A. H. Mekhemer, Mohamed-Elamir F. Hegazy, Yanshuo Han, Tarad Abalkhail

**Affiliations:** ^1^ Chemistry Department, Faculty of Science, Minia University, Minia, 61519, Egypt, minia.edu.eg; ^2^ School of Health Sciences, University of KwaZulu-Natal, Westville Campus, Durban, 4000, South Africa, ukzn.ac.za; ^3^ Department of Supportive Requirements, University of Technology and Applied Sciences, Nizwa, 611, Oman, hct.edu.om; ^4^ Department of Pharmacognosy, Faculty of Pharmacy, Ain Shams University, Cairo, 11566, Egypt, asu.edu.eg; ^5^ Department of Microbial Drugs, Helmholtz Centre for Infection Research GmbH, Braunschweig, 38124, Germany; ^6^ Department of Pharmaceutical Chemistry, Faculty of Pharmacy, Tanta University, Tanta, 31527, Egypt, tanta.edu.eg; ^7^ Chemistry of Medicinal Plants Department, National Research Centre, Giza, 12622, Egypt, nrc.sci.eg; ^8^ School of Life and Pharmaceutical Sciences, Dalian University of Technology, Panjin, China, dlut.edu.cn; ^9^ Department of Botany and Microbiology, College of Science, King Saud University, Riyadh, 11451, Saudi Arabia, ksu.edu.sa

**Keywords:** binding energy calculation, covalent docking, DprE1, molecular dynamics simulation, NPAtlas

## Abstract

As the second most deadly infectious disease worldwide after COVID‐19, tuberculosis (TB) remains a pressing global health issue, further aggravated by multidrug‐resistant TB (MDR‐TB) and extensively drug‐resistant TB (XDR‐TB) strains. There is an urgent need to identify new anti‐TB treatments and novel therapeutics to confront drug resistance. The decaprenylphosphoryl‐D‐ribose oxidase (DprE1) is an essential protein for the biosynthesis of the mycobacterial cell wall, and its inhibition features a promising antitubercular strategy. NPAtlas was utilized as a reference database, comprising natural products with confirmed biological effects. The aim of the current study is to identify and prioritize promising nitro‐containing natural products from the NPAtlas as potential covalent DprE1 inhibitors using advanced in silico approaches. Herein, the docking scores of 133 nitro‐containing NPAtlas compounds were assessed using a covalent docking technique. Thereafter, NPAtlas compounds with docking scores lower than PBTZ169 (calc. −7.8 kcal·mol^−1^) were subjected to molecular dynamics simulation (MDS), accompanied by binding energy estimations utilizing the MM‐GBSA approach. Based on MM‐GBSA//250 ns MDS, NPA011203, NPA013234, NPA016048, NPA012944, NPA001712, and NPA002823 demonstrated higher binding affinities against DprE1 with Δ*G*_binding_ values of −75.6, −62.7, −61.6, −57.6, −54.8, and −50.7 kcal·mol^−1^, respectively, than PBTZ169 (calc. −49.4 kcal·mol^−1^). The identified NPAtlas compounds also demonstrated structural and energetic stability within the DprE1 active site throughout 250 ns MDS. Physicochemical and ADMET predictions of the identified NPAtlas compounds indicated a suitable molecular size, favorable absorption, and negligible toxicity, suggesting their potential oral bioavailability. These in silico outcomes provide preliminary insights into the identified NPAtlas compounds as potential DprE1 inhibitors and can guide subsequent in vitro/in vivo experiments.

## 1. Introduction

Tuberculosis (TB) is a respiratory disease caused by a closely related group of bacteria known as the *Mycobacterium tuberculosis* complex (MTC or MTBC) [1]. This complex comprises several species, including *Mycobacterium tuberculosis*, *Mycobacterium africanum*, *Mycobacterium canettii*, *Mycobacterium bovis*, and *Mycobacterium orygis*, with MTC being the predominant pathogen responsible for TB in humans [2–6]. The primary infectious agent, MTC, spreads mainly through airborne droplets and has a strong tendency to infect the lungs [7, 8]. According to the World Health Organization (WHO), TB causes approximately 1.3–1.6 million deaths annually and affects nearly 16 million people worldwide [9]. The emergence of multidrug‐resistant TB (MDR‐TB), extensively drug‐resistant TB (XDR‐TB), and highly drug‐resistant strains has heightened the urgent need for effective control measures [10, 11]. The Global Tuberculosis Report 2020 documented around 437,000 cases of MDR‐TB, underscoring the necessity for innovative therapeutic strategies [10, 12]. In recent years, both computational and experimental approaches have contributed significantly to understand the complex molecular mechanisms of TB and have become instrumental in identifying potential drug targets [13–16]. Identifying new anti‐TB treatments and novel therapeutic targets is urgently needed to prevent resistance. In this regard, decaprenylphosphoryl‐D‐ribose oxidase (DprE1) has been identified as a promising target for novel antitubercular drugs due to its crucial role in mycobacterial cell wall biosynthesis [17, 18]. DprE1 is also critical in the final stages of arabinan biosynthesis, a process vital for maintaining the structural integrity and virulence of MTC [19]. Inhibition of DprE1 can minimize human cell toxicity, as MTC features unique metabolic and cell envelope pathways [19]. The diverse mechanisms of DprE1 inhibition, including both covalent and noncovalent approaches, provide significant potential for discovering new antitubercular agents [20, 21]. The current landscape of clinical investigations includes four inhibitors targeting DprE1, namely BTZ043, OPC167832, TBA7371, and PBTZ169 (macozinone) [22]. OPC167832 and TBA7371, classified as noncovalent inhibitors, exhibit potent antimycobacterial activity [23–25]. On the other hand, BTZ043 and PBTZ169 are covalent‐specific DprE1 inhibitors within the nitrobenzothiazinones (BTZs) class. The BTZ class of compounds has yielded promising anti‐TB agents, with BTZ043 being one of the earliest and most effective. PBTZ169 demonstrated superior potency, with an MIC of 0.2 ng/mL, establishing it as a leading candidate for TB treatment [26]. BTZs are categorized as mechanism‐based inhibitors that result in the irreversible inactivation of DprE1 [19, 27, 28]. As nitro‐containing compounds, BTZs serve as masked electrophiles capable of creating an irreversible bond with the CYS387 residue in DprE1 [27, 29–31]. From prior studies, it is evident that PBTZ169 offers several advantages over BTZ043, including cost‐effectiveness, a simplified synthesis route, and an enhanced pharmacodynamic profile [32]. Preclinical models have demonstrated the synergistic effects of PBTZ169 in combination with clofazimine and bedaquiline [33]. Phase I clinical trials evaluating PBTZ169 for TB treatment were completed in Switzerland, and Phase II trials are currently underway in Russia [34]. Recent scientific studies have shown that PBTZ169 significantly enhances patient survival rates in MDR‐TB cases, highlighting its potential as a viable treatment option for individuals who do not respond adequately to conventional TB drugs [35, 36]. However, mutations within the DprE1 active site, particularly those involving the catalytic cysteine residue (CYS387), have been reported to confer resistance to covalent inhibitors [37]. Notably, the U.S. Food and Drug Administration (FDA) has not yet approved any medicinal substance targeting DprE1, underscoring the motivation behind the present study to discover covalent inhibitors for this formidable pathogen [38].

Earlier efforts focused on identifying potent DprE1 inhibitors using virtual screening of large chemical databases [17, 39]. Nature has played a vital role in modern drug discovery, with approximately 40% of FDA‐approved drugs originating from natural products (NPs) or their structural derivatives [40]. Consequently, NPs have attracted considerable attention as a rich source of prospective therapeutics for the treatment of various diseases [41]. However, the lengthy timelines and high costs associated with the extraction and experimental screening of NPs have prompted the development of curated chemical libraries consisting of naturally derived compounds [42]. Among these resources, the Natural Products Atlas (NPAtlas) database is one of the most widely used repositories for microbially derived NPs and their associated bioactivity data [43].

**FIGURE 1:**
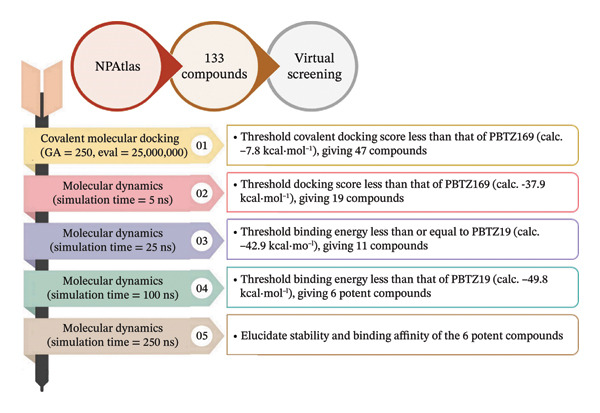
Graphical overview of the applied in silico workflow for screening NPAtlas compounds against DprE1. The protocol comprises an initial covalent docking step (orange), followed by a stepwise molecular dynamics simulation (MDS) refinement strategy, including initial (5 ns, pink), intermediate (25 ns, purple), advanced (100 ns, green), and final long‐timescale MDS (250 ns, beige).

To this end, 133 nitro‐containing NPAtlas compounds were computationally screened against DprE1 using advanced in silico techniques, including covalent docking predictions and molecular dynamics simulations (MDSs), followed by binding energy computations using the MM‐GBSA approach. All NPAtlas‐derived compounds were compared to PBTZ169 as a reference compound. It is worth mentioning that PBTZ169 is known to function through a unique protein‐catalyzed covalent mechanism; nevertheless, this study cannot verify that the identified nitro‐containing NPAtlas compounds, as DprE1 inhibitors, follow this exact mechanism. Accordingly, the identified NPAtlas compounds, proposed as potential covalent inhibitors, warrant experimental validation to confirm their postulated mechanism of action. Figure 1 provides a visual representation of the in silico approaches used to screen the NPAtlas compounds against DprE1. This study presents the first in silico assessment of nitro‐containing NPAtlas compounds targeting DprE1, aiming to identify promising covalent inhibitors. Notably, computational approaches inevitably simplify biological systems and cannot fully capture the complexity of living organisms, including processes such as cellular uptake, metabolism, off‐target interactions, and immune responses. Accordingly, the principal limitation of this study is the lack of experimental validation of the proposed DprE1 inhibitors, underscoring the necessity for subsequent in vitro and in vivo investigations to better assess the therapeutic potential of the identified NPAtlas compounds against TB disease.

## 2. Computational Methods

### 2.1. DprE1 Preparation

A high‐resolution structure of DprE1 in complex with PBTZ169 was obtained from the Protein Data Bank (PDB), accessed via the Research Collaboratory for Structural Bioinformatics (RCSB) website (PDB ID: 4NCR; resolution: 1.88 Å) [33]. To ensure structural integrity, missing residues were computationally modeled using the Modeller software [44]. Nonessential structural components, such as water molecules, heteroatoms, the cocrystallized inhibitor, and an additional protein chain, were removed to prevent unwanted interactions during docking computations. Furthermore, all missing hydrogen atoms were added using the H++ web server [45]. To evaluate the pKa values of DprE1 residues, H++ calculations were conducted under physiological conditions with an external dielectric constant of 80, an internal dielectric constant of 10, pH of 7.0, and a salinity of 0.15. Notably, the protonation states of key catalytic and active‐site residues in DprE1, particularly CYS387 and neighboring residues, were manually inspected to ensure chemically reasonable states prior to covalent docking computations.

### 2.2. Covalent Inhibitors Preparation

To identify potential DprE1 covalent inhibitors, an extensive exploration was performed within the NPAtlas database, yielding 133 nitro‐containing compounds. The 2D representations of NPAtlas compounds were provided in SDF format [46]. To facilitate further analysis, the 2D NPAtlas structures were converted into 3D conformations using Omega software [47, 48]. The resulting 3D NPAtlas structures underwent energy minimization employing the MMFF94S force field within the SZYBKI software [49–51]. Partial atomic charges of the NPAtlas compounds were computed employing the Gasteiger‐Marsili method [52]. The FixpKa program, embedded within the QUACPAC software, was employed to determine the most probable ionization status of the molecules [53]. A schematic flow diagram summarizing the compound preparation workflow is shown in Figure S1.

### 2.3. Covalent Docking

Covalent docking is an advanced computational approach that predicts selective inhibitor binding by simulating both noncovalent interactions and covalent bond formation, thereby facilitating the identification of potent, specific, and durable therapeutic candidates [54]. The AutoDock 4.2.6 software was used to perform covalent docking computations against DprE1, employing the flexible side‐chain methodology [55, 56]. This method was chosen because of the action of several known DprE1 inhibitors, such as PBTZ169, which act through covalent bond formation with a key active‐site residue, CYS387. Using this approach enables more precise modeling of inhibitor‐protein interactions relevant to DprE1 inhibition. Before conducting the covalent docking computations, the DprE1 structure was converted into the pdbqt format, a standardized file format that facilitates efficient docking analyses [57]. Most covalent docking parameters were set to their default values, except that the number of genetic algorithm (*GA*) runs was increased to 250, and the maximum number of energy evaluations (*eval*) was increased to 25,000,000. A grid box with dimensions of 40 Å × 40 Å × 40 Å, centered at *x* = 17.176, *y* = −20.119, and *z* = 1.875, and a spacing value of 0.375 Å was constructed around the DprE1 active site to ensure accurate binding predictions.

### 2.4. MDS

To gain insights into the interaction between the most probable NPAtlas compounds and DprE1, MDS was conducted using the AMBER20 software, a widely used and validated package for biomolecular simulations [58]. Specific details of the MD setup, such as the force field parameterization and the treatment of DprE1, were previously disclosed in [59–61]. General AMBER force field (GAFF2) was employed for parameterizing NPAtlas compounds [62]. DprE1 was characterized using the AMBER force field of 14SB, a well‐established force field for proteins [63]. To assign atomic charges, the irreversible covalent inhibitors in complex with the CYS387 residue were capped using N‐terminal acetyl and C‐terminal methyl amide groups. Afterward, these capped systems were subjected to optimization at the B3LYP/6‐31G^∗^ level, utilizing the Gaussian 09 software [64]. The atomic charges of the NPAtlas compounds were determined using the restrained electrostatic potential (RESP) approach at the HF/6‐31G^∗^ level, a well‐established method for charge derivation [65]. Furthermore, the investigation involved utilizing the antechamber module, an integral component of the AMBER20 package, to characterize and establish the atom types and parameters associated with covalent NPAtlas compounds interacting with the CYS387 residue. The NPAtlas‐DprE1 complexes were placed in an octahedral simulation box containing TIP3P water molecules, maintaining an average distance of 12 Å from the edges of the solute. To maintain system neutrality, counter ions (Na^+^/Cl^−^) were introduced, and a concentration of 0.15 M NaCl, a common physiological salt concentration, was reached [66]. The solvated complexes underwent a 5000‐step minimization process, followed by gradual heating to 310 K over 50 ps. Equilibration was then carried out for 10 ns to attain stability in the complexes. Following equilibration, production stages were sequentially extended from 5 ns to 25 ns, 100 ns, and finally 250 ns. During these simulations, trajectory data were recorded at regular intervals of 10 ps, a time step commonly used in similar computational studies. Bonds involving hydrogen atoms were constrained employing the SHAKE algorithm, allowing for a 2fs integration time step [67]. Long‐range electrostatic interactions were modeled using the particle mesh Ewald (PME) summation method, employing a cutoff distance of 12 Å [68]. Pressure and temperature were maintained using the Berendsen barostat and Langevin thermostat, respectively [69, 70]. MDS was conducted using the PMEMD.CUDA GPU feature integrated into the AMBER20 software. Furthermore, inhibitor‐DprE1 interactions and visualization were performed through the BIOVIA Discovery Studio platform [71].

### 2.5. MM‐GBSA Binding Energy

The binding energy (Δ*G*_binding_) of the investigated NPAtlas‐DprE1 complexes was computed employing the molecular mechanics‐generalized Born surface area (MM‐GBSA) approach [72]. Polar solvation energy was estimated utilizing the GB model (igb = 2) proposed by Onufriev et al. [73]. In accordance with the given equation, the binding energy (Δ*G*_binding_) was calculated as follows:

(1) ΔGbinding=GComplex−GDprE1+GNPAtlasCompound.

Here, the energy term (*G*) was calculated using the following equation:

(2) G=Gsolv+EMM−TS,

(3) EMM=EvdW+Eele+Eint,

(4) Eint=Eangle+Ebond+Etorsion,

where *E*_MM_ refers to the energy of the gas‐phase molecular mechanics (MM) calculations. *G*_solv_ corresponds to the solvation energy. *E*_vdW_ specifically indicates the van der Waals energy. *E*_ele_ points out the electrostatic energy. *E*_int_ denotes the internal energy, incorporating contributions from angle, bond, and dihedral energies. The spatial coordinates of the covalent NPAtlas compounds, DprE1, and the NPAtlas‐DprE1 complexes were obtained using a single‐trajectory technique. Entropy contributions were omitted to reduce computational cost, simplifying the calculations [74, 75]. It is noteworthy that neglecting the entropic contribution would not result in any significant change in the binding energy computations [76].

### 2.6. Drug‐Likeness Features

Drug‐likeness properties have been proposed as a means of filtering out compounds with undesired characteristics in drug‐discovery processes [77–79]. A widely used guideline is Lipinski’s rule of five (Ro5), which establishes threshold criteria for key physicochemical properties that influence the oral bioavailability (BA) of compounds [80]. According to Ro5, a compound must meet the following criteria to be considered drug‐like: molecular weight (MW) ≤ 500 Da, octanol‐water partition coefficient (Mlog *p*) ≤ 5, number of H‐bond acceptors (HBAs) ≤ 10, number of H‐bond donors (HBDs) ≤ 5, and topological polar surface area (TPSA) ≤ 140 Å^2^. The prediction of drug‐likeness features was performed using the SWISS‐ADME server (https://www.swissadme.ch).

### 2.7. ADMET Profiles

The ADMET properties, including chemical absorption, distribution, metabolism, excretion, and toxicity, play a crucial role in verifying the therapeutic efficacy of potential drug candidates [81]. The assessment of ADMET properties for the most active NPAtlas compounds against DprE1 was conducted using the pkCSM web‐based tool (https://biosig.unimelb.edu.au/pkcsm/prediction). Critical assessments included the estimation of water solubility permeability (log *S*) and skin permeability (log *Kp*) to evaluate NPAtlas compounds absorption. The distribution was estimated according to blood‐brain barrier (BBB) penetration by calculating log BB, and central nervous system (CNS) permeability by estimating log PS. Metabolism was examined using Cytochrome P450 (CYP) substrate and inhibitor models, specifically CYP3A4 and CYP2D6, whereas excretion was determined by total clearance estimations. Toxicity evaluations included AMES toxicity, hERG I and II inhibition, oral rat acute toxicity (LD50), hepatotoxicity, and skin sensitization.

## 3. Results and Discussion

### 3.1. Covalent Docking for Nitro‐Containing NPAtlas Compounds

The ability of the covalent docking protocol using AutoDock4.2.6 software to accurately predict inhibitor‐DprE1 binding modes was previously validated [59, 60]. Minutely, redocking of the reference inhibitor PBTZ169 into the DprE1 active site accurately reproduced the experimental binding pose with an RMSD of 0.73 Å, demonstrating the reliability of the employed docking protocol [59, 60]. To further substantiate its reliability, five experimentally established irreversible DprE1 inhibitors, namely BTZ043, DNB1, VI‐9376, BTO, and cBT, with reported MIC values, were previously evaluated and demonstrated docking scores consistent with experimental findings, giving a correlation coefficient (*R* ^2^) value of 0.93 [59]. Following validation, a library of 133 nitro‐containing NPAtlas compounds was then screened, with docking scores tabulated in Table S1. Interestingly, out of these 133 NPAtlas compounds, 47 demonstrated covalent docking scores lower than that of PBTZ169 (calc. −7.8 kcal·mol^−1^). Figure S2 provides 2D representations for the most probable 47 NPAtlas compounds complexed with DprE1. Upon examining Figure S2, it can be observed that most NPAtlas compounds exhibited similarity in their docking poses against DprE1, establishing significant H‐bonds with TRP17, THR118, HIS132, GLN334, VAL365, and LYS418, thereby achieving favorable docking scores. Table 1 provides valuable information about the 2D chemical structures, computed covalent docking scores, and binding features of the top six probable NPAtlas inhibitors complexed with DprE1. Remarkably, these six NPAtlas compounds were selected based on the computed MM‐GBSA binding energy over a 250 ns MDS, which will be discussed in detail in the subsequent sections.

**TABLE 1:** 2D chemical structures, covalent docking scores (in kcal·mol^−1^), and key binding features for the top six predicted NPAtlas compounds and PBTZ169 against DprE1.

No.	NPAtlas code	2D chemical structure	Covalent docking score (kcal·mol^−1^)	Binding features^a^
	PBTZ169 (macozinone)	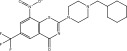	−7.8	LYS134 (2.52 Å), LYS418 (2.00 Å)
1	NPA011203	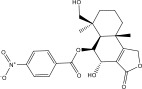	−15.8	TYR60 (1.99 Å), GLY117 (2.27 Å), VAL365 (1.80 Å), ASN385 (2.82 Å)
2	NPA013234	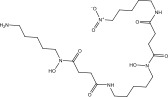	−15.3	GLY117 (2.93 Å), THR118 (3.13 Å), HIS132 (2.52 Å), GLN336 (3.06 Å), LYS418 (1.90, 1.94, 1.97 Å)
3	NPA016048	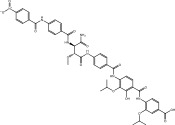	−15.1	TRP17 (2.48 Å), ARG58 (2.03, 2.23 Å), THR118 (2.22 Å), ASN385 (1.84 Å), VAL388 (1.79 Å), LYS418 (1.95, 2.67, 2.73 Å)
4	NPA012944	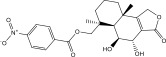	−14.7	TRP17 (2.45 Å), GLY117 (2.28 Å), TYR327 (2.10 Å)
5	NPA001712	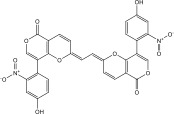	−14.4	TRP17 (3.25 Å), HIS132 (2.98 Å), GLN334 (1.67, 2.28 Å), VAL365 (1.79 Å)
6	NPA002823	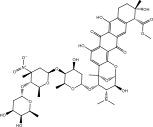	−13.9	TRP17 (1.82 Å), GLN120 (1.99, 2.99 Å), GLY133 (2.25 Å), ASP318 (1.81 Å), ALA330 (1.98 Å), GLN334 (2.36, 2.67 Å), GLN336 (3.53 Å), VAL365 (2.69 Å)

^a^Only intermolecular H‐bonds with key DprE1 active site residues are presented.

Figure 2 showcases 3D representations of NPA011203, NPA013234, NPA016048, NPA012944, NPA001712, and NPA002823 within the active site of DprE1. Of particular interest, the NO_2_ group of these six remarkable compounds forms an irreversible covalent bond with the SH group of CYS387, with bond lengths around 1.81 Å (Figure 2).

FIGURE 23D molecular interactions of (a) NPA011203, (b) NPA013234, (c) NPA016048, (d) NPA012944, (e) NPA001712, and (f) NPA002823 against DprE1. Atom color coding is as follows: carbon (grey), nitrogen (blue), oxygen (red), sulfur (yellow), and hydrogen (white). This figure was generated using BIOVIA Discovery Visualizer.

**Figure:**
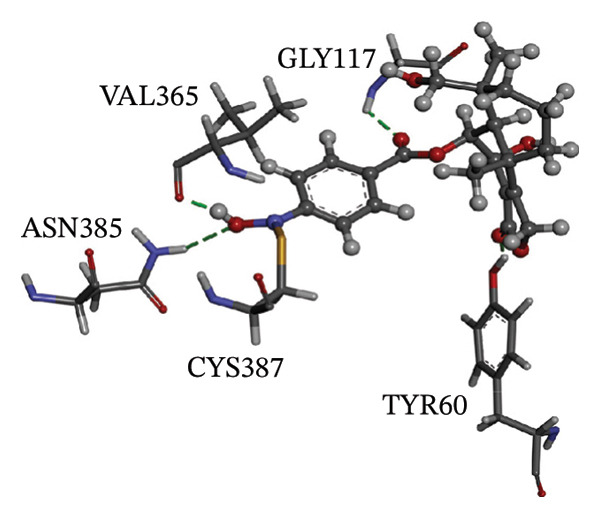
(a)

**Figure:**
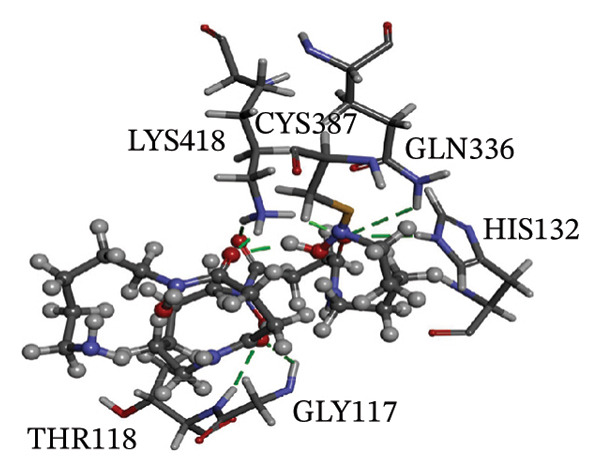
(b)

**Figure:**
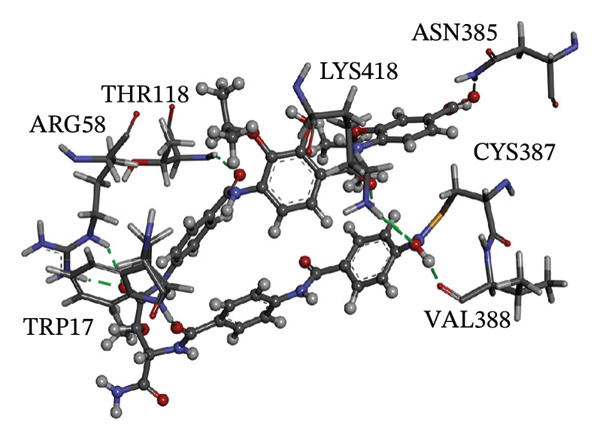
(c)

**Figure:**
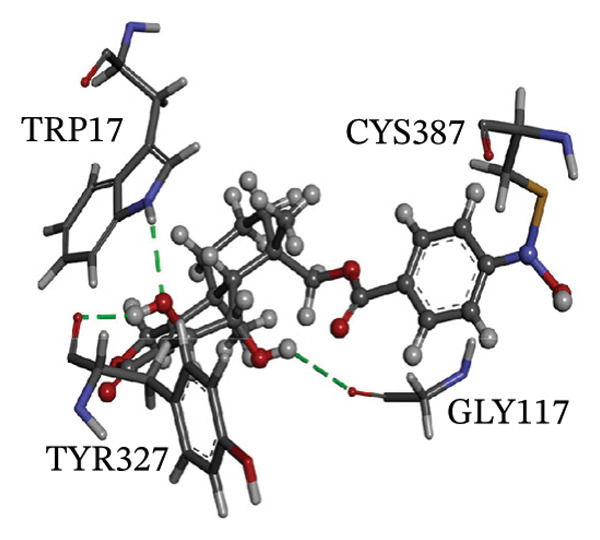
(d)

**Figure:**
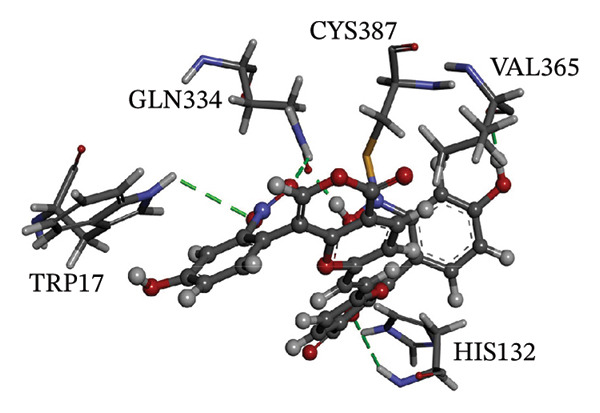
(e)

**Figure:**
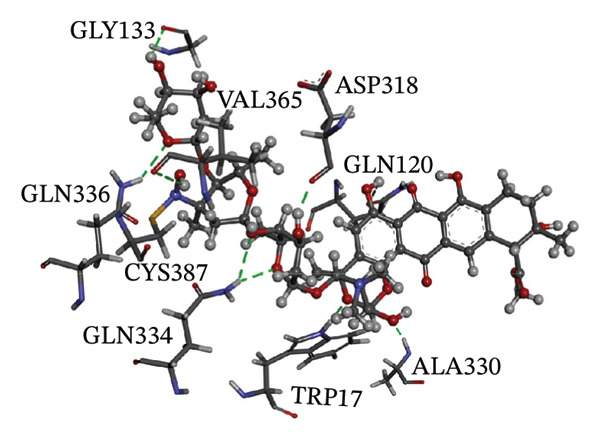
(f)

NPA011203, identified as 9*α*,14‐dihydroxy‐6*β*‐*p*‐nitrobenzoylcinnamolide, has emerged as a compelling cytotoxic agent, displaying significant efficacy against HCT‐116 human colon carcinoma cells [82]. NPA011203, as highlighted in Table 1, was identified as a probable candidate with a remarkable affinity for DprE1, as indicated by its covalent docking score of −15.8 kcal·mol^−1^. Intriguingly, the docking analysis revealed the formation of four H‐bonds between NPA011203 and key residues within the DprE1 active site, as summarized in Table 1. Notably, the nitro group of NPA011203 exhibited an H‐bond with the carbonyl group of VAL365 (1.80 Å), representing one of the critical interactions contributing to stable binding (Figure 2).

NPA013234 demonstrated the second‐highest binding affinity for DprE1, achieving a covalent docking score of −15.3 kcal·mol^−1^. The robust binding interaction between NPA013234 and DprE1 may be attributed to the formation of seven H‐bonds with the key residues inside the active site of DprE1. For example, the carbonyl oxygen of 18‐oxo‐6,11,17,22‐tetraazaheptacosyl amide, the carbonyl oxygen of 10‐oxo‐6,11,17,22‐tetraazaheptacosyl amide, and the nitro group contributed to three H‐bonds with the oxygen group and the NH_3_ of LYS418 (1.90, 1.94, and 1.97 Å) (Figure 2).

NPA016048, known as cystobactamide 919‐1 and classified as a myxobacterial topoisomerase inhibitor, exhibits promising antibacterial activity [83]. More significantly, NPA016048 demonstrated a third superior binding affinity toward DprE1, elucidated by a substantial covalent docking score of −15.1 kcal·mol^−1^. Notably, this compound formed a total of nine H‐bonds with the key residues within the DprE1 active site, as summarized in Table 1. For instance, the carbonyl oxygen of 4‐carboxy‐2‐isopropoxyphenyl contributed to an H‐bond with the NH group of THR118 (2.22 Å) (Figure 2).

NPA012944, identified as a derivative originating from 9*α*,14‐dihydroxy‐6*β*‐*p*‐nitrobenzoylcinnamolide, has emerged as a captivating cytotoxic agent [82]. Notably, NPA012944 demonstrated a significant propensity for binding DprE1, as evidenced by an impressive covalent docking score of −14.7 kcal·mol^−1^. Following the data presented in Table 1, the hydroxyl group of 11‐dihydroxy formed two H‐bonds with the NH group of TRP17 (2.45 Å) and with the carbonyl group of TYR327 (2.10 Å) (Figure 2).

NPA001712, known as phomopsis‐H76 C, is a polyoxygenated aromatic metabolite isolated from the mangrove‐derived endophytic fungus *Phomopsis* sp. [84, 85] and unveiled a satisfactory covalent docking score with a value of −14.4 kcal·mol^−1^ toward DprE1 (Table 1). This good docking score may be ascribed to the establishment of five H‐bonds with the proximal residues within the DprE1 active site. Among them, the nitro group of NPA001712 exhibited an H‐bond with the carbonyl group of VAL365 (1.79 Å) (Figure 2).

NPA002823, known as cororubicin, belongs to the class of anthracycline antibiotics and exhibits the unique ability to produce active oxygen species within tumor cells [86]. As demonstrated in Table 1, NPA002823 demonstrated a good covalent docking score of −13.9 kcal·mol^−1^ toward DprE1. Additionally, NPA002823 formed ten H‐bonds with the critical residues within the DprE1 active site. For instance, the oxygen atom of methyltetrahydropyranol and the oxygen atom of bis(methyltetrahydropyran) contributed to two H‐bonds with the NH_2_ of GLN334 (2.36 and 2.67 Å) (Figure 2).

### 3.2. MDS

**FIGURE 3:**
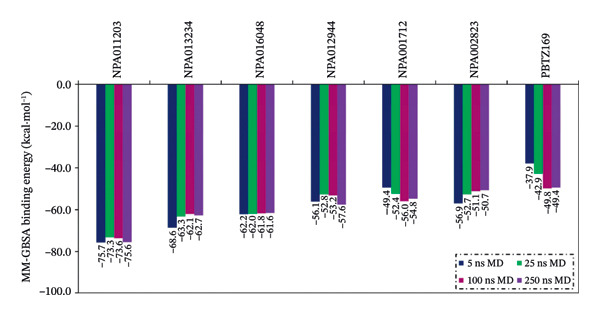
Binding energy estimations of the six probable NPAtlas compounds and PBTZ169 against DprE1 over 5, 25, 100, and 250 ns MDS.

MDS is employed as a robust computational tool to meticulously investigate the flexibility of key residues and assess the stability of the biological systems over time [87, 88]. In this study, MDS was employed to investigate the impact of DprE1 elasticity and conformational changes on the complexes formed between NPAtlas compounds and the DprE1 active site. Initially, 47 NPAtlas compounds with docking scores < −7.8 kcal·mol^−1^ were selected for MDS over 5 ns. The corresponding binding energies of these compounds complexed with DprE1 were then computed and are recorded in Table S2. From Table S2, 19 out of 47 NPAtlas compounds exhibited binding energies (Δ*G*_binding_) lower than PBTZ169 (calc. −37.9 kcal·mol^−1^) against DprE1. To ensure the reliability of the results, these 19 NPAtlas compounds complexed with DprE1 underwent 25 ns MDS, accompanied by binding energy estimations (Table S3). Based on the data presented in Table S3, 11 out of 19 NPAtlas compounds displayed Δ*G*_binding_ less than PBTZ169 (calc. −42.9 kcal·mol^−1^). To further confirm these findings, the 11 NPAtlas compounds complexed with DprE1 were subjected to extended 100 ns MDS. The corresponding binding energies were computed and are summarized in Table S4. Remarkably, approximately 5% of the screened NPs (i.e., six NPAtlas compounds) demonstrated superior binding energies against DprE1, compared to PBTZ169 (Δ*G*_binding_ = −49.8 kcal·mol^−1^) (Table S4). Consequently, the MDS was elongated to 250 ns for NPA011203, NPA013234, NPA016048, NPA012944, NPA001712, and NPA002823 in complex with DprE1. The binding energies of these NPAtlas compounds were computed over the 250 ns MDS and are depicted in Figure 3.

Examination of the bar chart in Figure 3 revealed no significant variation in the evaluated binding energies of the identified NPAtlas compounds between 100 and 250 ns MDS. Compared to PBTZ169 (calc. −49.4 kcal·mol^−1^), NPA011203, NPA013234, NPA016048, NPA012944, NPA001712, and NPA002823 exhibited remarkable binding energies with DprE1 throughout 250 ns MDS, with Δ*G*_binding_ values of −75.6, −62.7, −61.6, −57.6, −54.8, and −50.7 kcal·mol^−1^, respectively. The presented findings emphasized the promising potential of the identified NPAtlas compounds as lead compounds for advancing anti‐TB pharmacotherapy.

**FIGURE 4:**
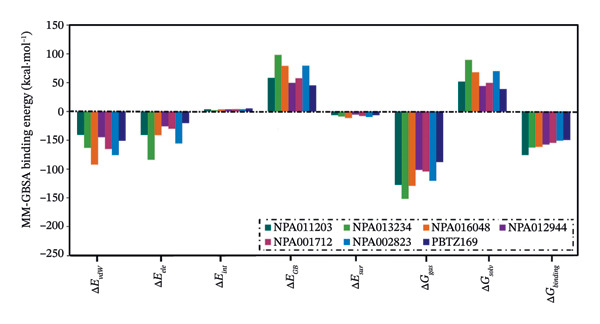
MM‐GBSA binding energy decomposition of NPA011203, NPA013234, NPA016048, NPA012944, NPA001712, NPA002823, and PBTZ169 against DprE1 through 250 ns MDS. The energy components include van der Waals interactions (Δ*E*_vdW_), electrostatic interactions (Δ*E*_ele_), internal energy (Δ*E*_int_), generalized Born polar solvation energy (Δ*E*_GB_), nonpolar solvation (surface) energy (Δ*E*_sur_), gas‐phase interaction energy (Δ*G*_gas_ = Δ*E*_vdW_ + Δ*E*_ele_), solvation energy (Δ*G*_solv_ = Δ*E*_GB_ + Δ*E*_sur_), and the total binding energy (Δ*G*_binding_).

In addition, an MM‐GBSA binding energy decomposition was conducted to elucidate the inherent driving forces controlling the binding of the identified NPAtlas compounds and PBTZ169 with DprE1 over 250 ns MDS (Figure 4). As depicted in Figure 4, the Δ*E*_vdW_ energies of NPA011203, NPA013234, NPA016048, NPA012944, NPA001712, NPA002823, and PBTZ169 dominated their interaction with DprE1 with values of −40.6/−169.8, −63.4/−265.1, −92.2/−385.7, −44.7/−186.9, −65.1/−272.5, −75.5/−316.0, and −51.0/−213.6 kcal·mol^−1^/kJ·mol^−1^, respectively. NPA011203, NPA013234, NPA016048, NPA012944, NPA001712, NPA002823, and PBTZ169 also exhibited favorable Δ*E*_ele_ energies with values of −40.8/−170.8, −84.0/−351.5, −41.2/−172.3, −25.8/−107.8, −30.0/−125.5, −55.8/−233.4, and −20.2/−84.4 kcal·mol^−1^/kJ·mol^−1^, respectively. The aforementioned findings present detailed statistical information on the binding energies of the identified NPAtlas compounds as putative DprE1 inhibitors.

**FIGURE 5:**
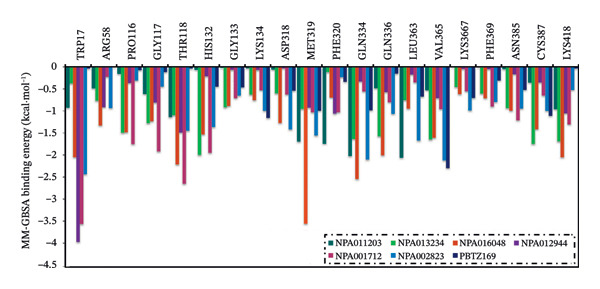
Decomposition per‐residue of NPA011203, NPA013234, NPA016048, NPA012944, NPA001712, NPA002823, and PBTZ19 against DprE1 through 250 ns MDS. key interacting residues: TRP17, THR118, MET319, VAL365, CYS387, and LYS418.

In order to examine NPAtlas‐DprE1 interactions and the contribution of proximal amino acids inside the DprE1 active site, estimated Δ*G*_binding_ values were decomposed into individual residue contributions (Figure 5). Figure 5 depicts the residues that significantly contribute (<−0.5 kcal·mol^−1^) to the binding of the identified NPAtlas compounds with DprE1. Residues such as TRP17, THR118, HIS132, MET319, GLN334, VAL365, CYS387, and LYS418 dominated the interactions between the identified NPAtlas compounds and DprE1 (Figure 5). As an example, VAL365 exhibited a substantial contribution to the total Δ*G*_binding_ with values of −0.5, −1.6, −1.6, −0.7, −0.9, −2.1, and −2.3 kcal·mol^−1^ for NPA011203, NPA013234, NPA016048, NPA012944, NPA001712, NPA002823, and PBTZ169, respectively.

**FIGURE 6:**
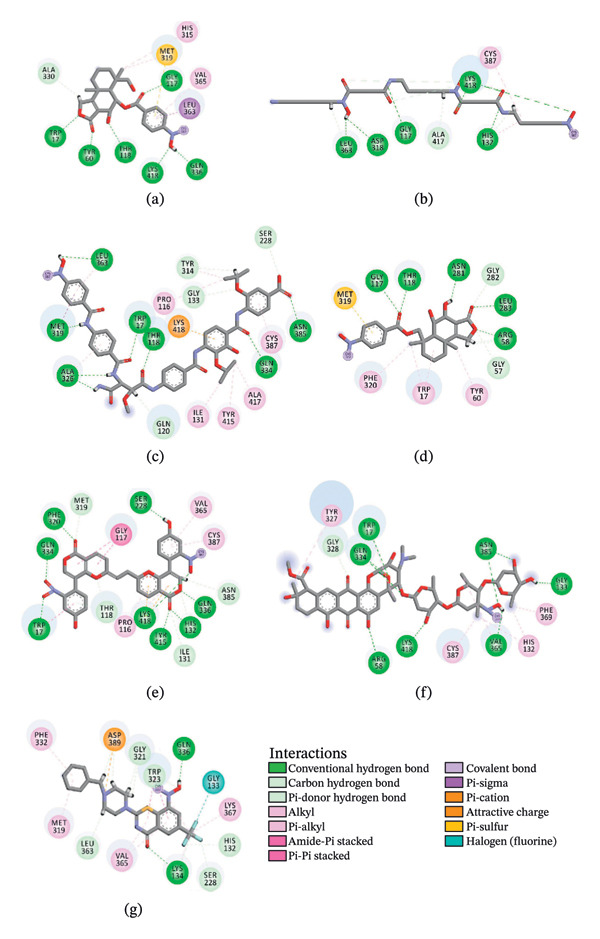
2D representations of the predicted binding modes of (a) NPA011203, (b) NPA013234, (c) NPA016048, (d) NPA012944, (e) NPA001712, (f) NPA002823, and (g) PBTZ169 with DprE1 from the final snapshot of a 250 ns MDS. The diagrams illustrated key interactions, including H‐bonds, hydrophobic contacts, π‐π interactions, and electrostatic interactions between the inhibitors and active site residues. All interaction maps were generated using BIOVIA Discovery Visualizer.

Besides, Figure 6 provides a visual 2D representation of the final trajectories of MDS for NPA011203, NPA013234, NPA016048, NPA012944, NPA001712, and NPA002823 within the DprE1 active site. The examined NPAtlas‐DprE1 complexes exhibited stable conformations throughout the simulation period; concurrently, additional intermolecular interactions were observed. For example, NPA011203 formed a new H‐bond with GLN336 (2.14 Å), which was absent in the docked complex. Moreover, multiple snapshots were extracted at regular intervals along the MDS to further evaluate the conformational stability of the identified NPAtlas compounds within the active site of DprE1. The 2D representations of the predicted binding modes of these NPAtlas compounds inside the DprE1 active site are shown in Figures S3–S6. As evident from Figures S3–S6, the investigated NPAtlas compounds maintained persistent interactions within the DprE1 active site throughout the simulation time, supporting the formation of stable DprE1‐inhibitor complexes.

### 3.3. Post‐MD Analyses

#### 3.3.1. Binding Energy Per‐Frame

To assess the stability of the identified NPAtlas compounds and PBTZ169 complexed with DprE1, a correlation analysis was conducted between the binding energy and the simulation time over the 250 ns MDS (Figure 7(a)). The mean MM‐GBSA binding energies, reported as mean ± standard deviation (±SD) to reflect fluctuation errors, were −75.6 ± 6.8, −62.7 ± 5.1, −61.6 ± 4.7, −57.6 ± 5.9, −54.8 ± 6.1, and −50.7 ± 5.3 kcal·mol^−1^ for NPA011203, NPA013234, NPA016048, NPA012944, NPA001712, and NPA002823 complexed with DprE1, respectively, compared to −49.4 ± 5.2 kcal·mol^−1^ for PBTZ169. Importantly, all the identified complexes exhibited sustained stability throughout the MDS, providing valuable insights into the consistency and reliability of these NPAtlas‐DprE1 complexes.

FIGURE 7(a) Binding energy per‐frame, (b) CoM distances, and (c) RMSD of NPA011203 (dark cyan), NPA013234 (green), NPA016048 (burnt orange), NPA012944 (violet), NPA001712 (dark pink), NPA002823 (blue), and PBTZ169 (navy), toward DprE1 over the 250 ns MD course. Trajectory analyses were performed using the CPPTRAJ module of AMBER20.

**Figure:**
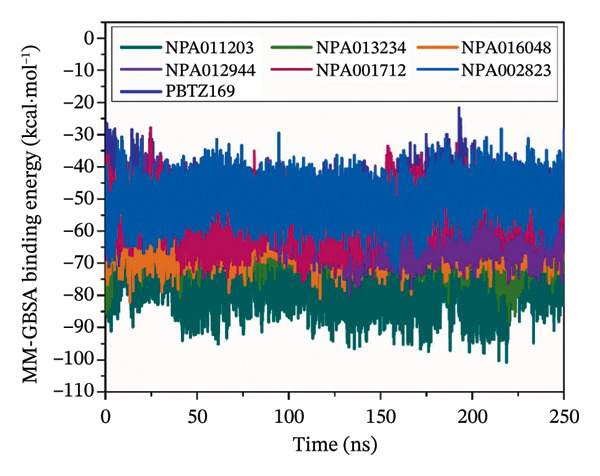
(a)

**Figure:**
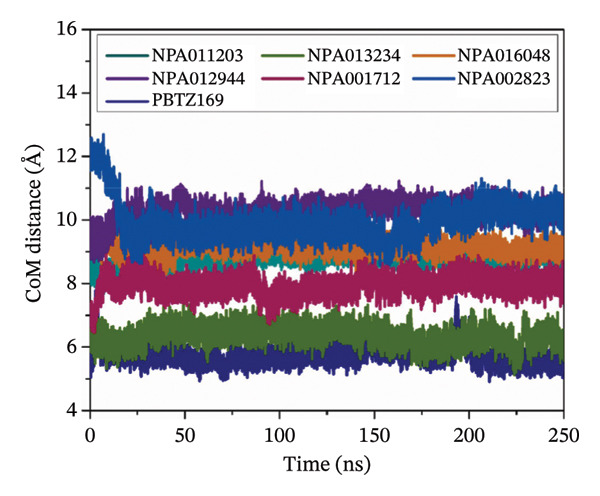
(b)

**Figure:**
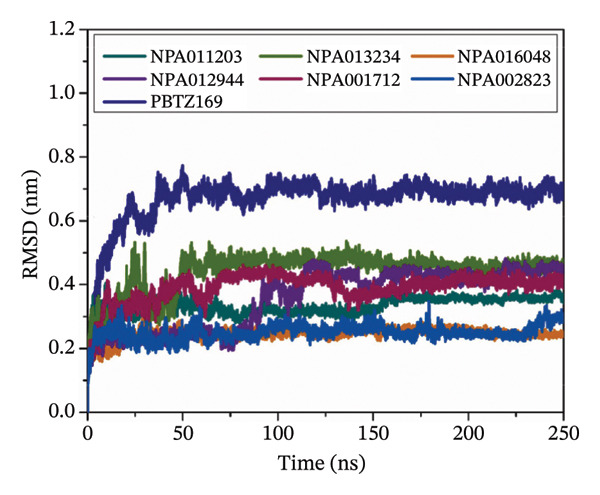
(c)

#### 3.3.2. Center‐of‐Mass (CoM) Distance

More insight into the consistency of NPAtlas‐DprE1 complexes was gained throughout 250 ns MDS by estimating the CoM distances between the identified NPAtlas compounds and CYS387 residue (Figure 7(b)). As shown in Figure 7(b), all investigated compounds exhibited minimal fluctuations and remained within a narrow range of the CoM distances throughout 250 ns MDS. Numerically, NPA011203, NPA013234, NPA016048, NPA012944, NPA001712, NPA002823, and PBTZ169 demonstrated average CoM distances of 8.76, 6.35, 9.13, 10.28, 7.94, 9.95, and 5.73 Å, respectively. To sum up, the CoM analyses revealed the high stability of the identified NPAtlas compounds within the DprE1 active site over 250 ns MDS.

#### 3.3.3. Root‐Mean‐Square Deviation (RMSD)

The conformational and positional variations of the identified NPAtlas compounds complexed with DprE1 were assessed through protein backbone RMSD analysis over 250 ns MDS (Figure 7(c)). As demonstrated in Figure 7(c), the mean RMSD values (±SD) for the NPA011203‐, NPA013234‐, NPA016048‐, NPA012944‐, NPA001712‐, NPA002823‐ and PBTZ169‐DprE1 were 0.33 ± 0.03, 0.44 ± 0.06, 0.25 ± 0.02, 0.35 ± 0.01, 0.39 ± 0.04, 0.25 ± 0.02, and 0.66 ± 0.08 nm, respectively. Importantly, the identified NPAtlas compounds exhibited low and stable RMSD values, indicating that their binding poses were well preserved within the DprE1 active site throughout 250 ns MDS. Notably, the PBTZ169‐DprE1 complex stabilized after 20 ns and remained stable until the end of the simulation, reflecting a structural adjustment rather than a loss of stability. Overall, these findings indicated that the identified NPAtlas compounds maintained robust and stable binding within the DprE1 active site.

#### 3.3.4. Root‐Mean‐Square Fluctuation (RMSF)

RMSF analysis of the C_α_ atoms was employed to understand the impact of the binding of the identified NPAtlas compounds on the structural fluctuations of DprE1 (Figure 8(a)). As illustrated in Figure 8(a), the average RMSF values for the apo‐, NPA011203‐, NPA013234‐, NPA016048‐, NPA012944‐, NPA001712‐, NPA002823‐, and PBTZ169‐DprE1 complexes were 0.14, 0.13, 0.14, 0.11, 0.13, 0.13, 0.12, and 0.14 nm, respectively. The fluctuation of DprE1 residues was displayed through elevated peaks in the RMSF analysis. Specific DprE1 residues, particularly in the 260–290 and 310–330 regions, exhibited significant fluctuations according to RMSF, indicating the NPAtlas compounds may introduce different restrictions on the motions around these residues. Based on the available evidence, the apo and soaked structures of DprE1 exhibited notable stability throughout the entire simulation. The variance observed in RMSF values reflected differences in the internal dynamics and interaction intensities of the protein‐inhibitor complexes, suggesting distinct stability and flexibility profiles among the systems studied.

FIGURE 8(a) RMSF, (b) Rg, and (c) SASA for apo‐DprE1 (magenta), NPA011203‐DprE1 (dark cyan), NPA013234‐DprE1 (green), NPA016048‐DprE1 (burnt orange), NPA012944‐DprE1 (violet), NPA001712‐DprE1 (dark pink), NPA002823‐DprE1 (blue), and PBTZ169‐DprE1 (navy) complexes during the 250 ns MDS.

**Figure:**
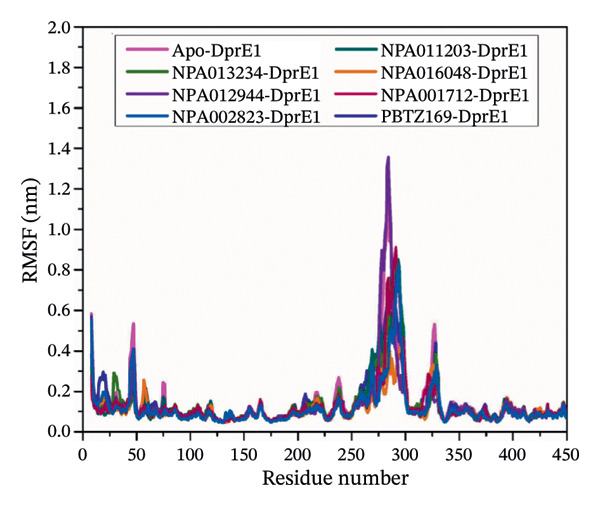
(a)

**Figure:**
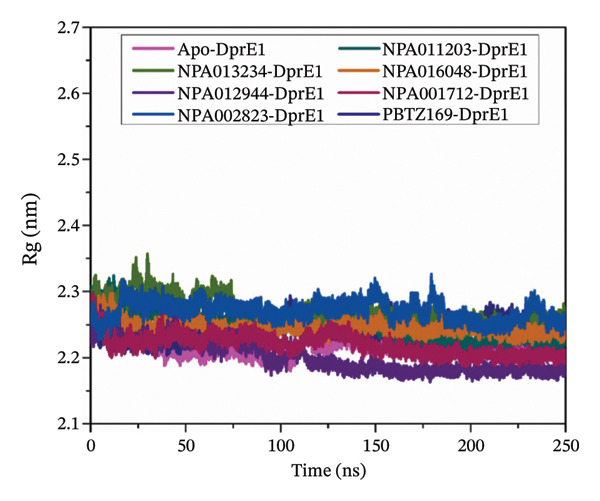
(b)

**Figure:**
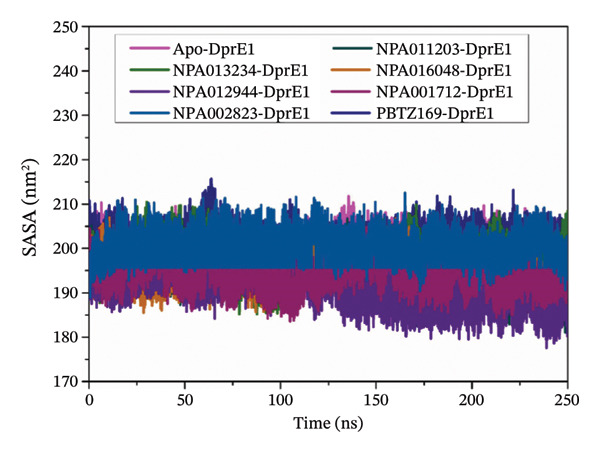
(c)

#### 3.3.5. Radius of Gyration (Rg)

The Rg was examined for the NPAtlas‐DprE1 complexes to investigate the impact of the NPAtlas compounds on the folding and unfolding dynamics of DprE1. The mean Rg values (±SD) for the apo‐, NPA011203‐, NPA013234‐, NPA016048‐, NPA012944‐, NPA001712‐, NPA002823‐, and PBTZ169‐DprE1 were 2.22 ± 0.01, 2.25 ± 0.02, 2.27 ± 0.02, 2.25 ± 0.01, 2.20 ± 0.02, 2.22 ± 0.01, 2.27 ± 0.01, and 2.24 ± 0.01 nm, respectively (Figure 8(b)). The negligible fluctuations noted in Rg indicated that DprE1 complexes maintained structural compactness and stability during the simulation, hence affirming the structural integrity of the NPAtlas‐DprE1 complexes.

#### 3.3.6. Solvent‐Accessible Surface Area (SASA)

To probe the effect of inhibitor binding on the solvent exposure of DprE1, SASA was monitored throughout 250 ns MDS. Figure 8(c) illustrates SASA analysis for apo‐, NPA011203‐, NPA013234‐, NPA016048‐, NPA012944‐, NPA001712‐, NPA002823‐, and PBTZ169‐DprE1. As illustrated in Figure 8(c), the mean SASA values (±SD) were 199.81 ± 3.15, 194.75 ± 3.99, 197.78 ± 3.69, 195.13 ± 3.03, 190.56 ± 4.20, 193.94 ± 3.20, 201.35 ± 3.08, and 202.06 ± 2.85 nm^2^ for the apo‐, NPA011203‐, NPA013234‐, NPA016048‐, NPA012944‐, NPA001712‐, NPA002823‐, and PBTZ169‐DprE1, respectively. The comparable SASA values indicated that binding of the NPAtlas compounds neither disrupts the overall conformation nor markedly alters the solvent accessibility of DprE1, confirming the structural stability of the complexes over 250 ns MDS.

#### 3.3.7. H‐Bond Number

**FIGURE 9:**
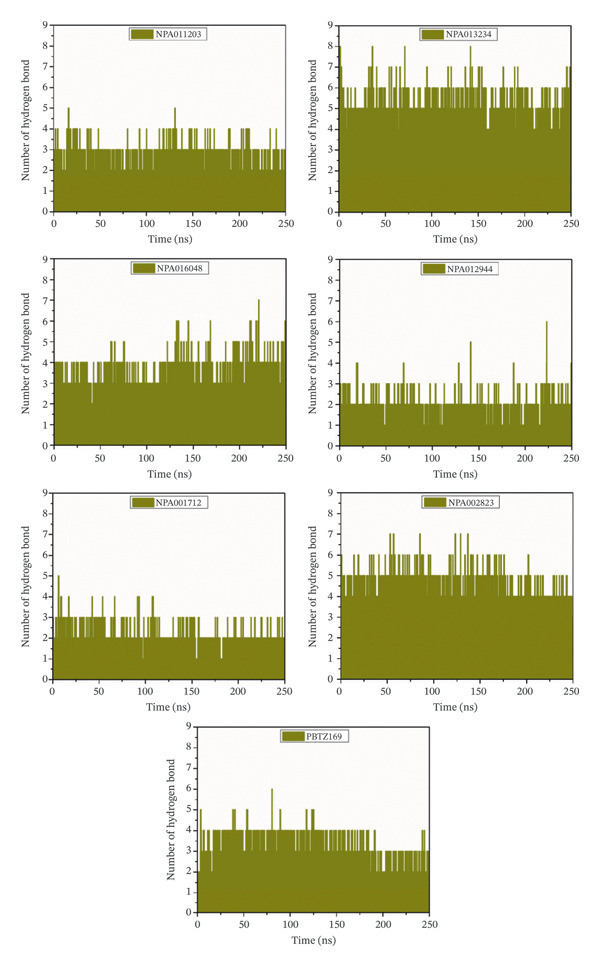
H‐bond number for NPA011203, NPA013234, NPA016048, NPA012944, NPA001712, NPA002823, and PBTZ169 complexed with DprE1 throughout 250 ns MDS.

To inspect the stability of the identified NPAtlas compounds complexed with DprE1, the number of intermolecular H‐bonds was measured over 250 ns MDS (Figure 9). On average, NPA011203, NPA013234, NPA016048, NPA012944, NPA001712, NPA002823, and PBTZ169 established approximately 3, 5, 3, 2, 2, 5, and 3 H‐bonds, respectively. Collectively, the persistence of these H‐bonding interactions underscores the strong and stable accommodation of the identified NPAtlas compounds within the DprE1 active site throughout the simulation time.

### 3.4. Drug‐Likeness Features

**FIGURE 10:**
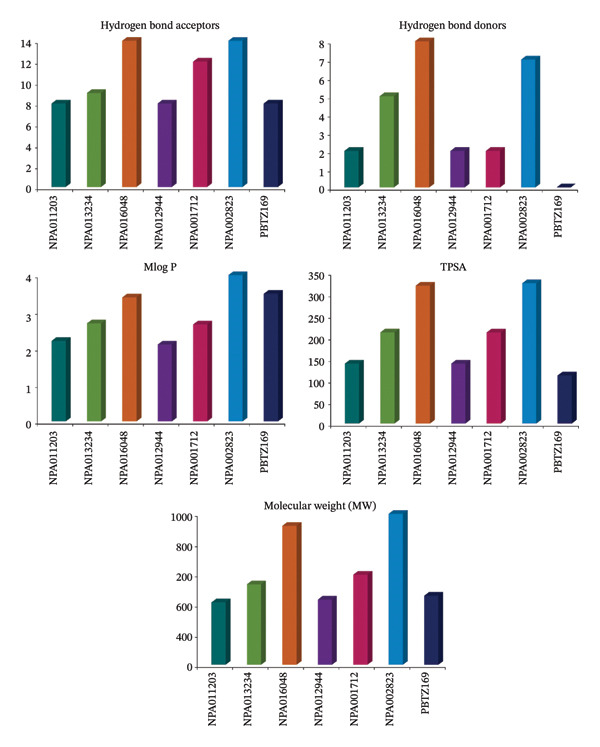
Computed drug‐likeness of NPA011203, NPA013234, NPA016048, NPA012944, NPA001712, NPA002823, and PBTZ169 as DprE1 inhibitors.

The exploration of drug‐likeness properties assumes a critical role during the initial stages of drug development, identifying compounds with favorable BA and potential efficacy against specific target proteins [89]. In this study, the investigation of drug‐likeness characteristics relied on applying the Ro5 using the SwissADME server (Figure 10). From the data illustrated in Figure 10, HBAs for NPA011203, NPA013234, NPA016048, NPA012944, NPA001712, NPA002823, and PBTZ169 were 8, 9, 14, 8, 12, 22, and 8, respectively. Besides, HBDs were 2, 5, 8, 2, 2, 7, and 0 for NPA011203, NPA013234, NPA016048, NPA012944, NPA001712, NPA002823, and PBTZ169, respectively. Notably, the Mlog *p* values for NPA011203, NPA013234, NPA016048, NPA012944, NPA001712, NPA002823, and PBTZ169 were found to be 2.2, 2.7, 3.4, 2.1, 2.7, 5.5, and 3.5, respectively, indicating their high lipophilicity. The measurements of the TPSA values revealed a range of 110.5–324.9 Å^2^. Moreover, the MWs of NPA011203, NPA013234, NPA016048, NPA012944, NPA001712, NPA002823, and PBTZ169 were determined to be 413.4, 532.6, 919.9, 431.4, 596.5, 1003.0, and 456.5 Da, respectively. Furthermore, additional drug‐likeness measures, including the number of rotatable bonds (RBs), synthetic accessibility (SA) score, and BA score, are provided in Table S5. As shown in Table S5, all investigated compounds displayed low RB counts and exhibited moderate SA and relatively favorable BA scores. Notably, the observed increases in MW and HBA count are unlikely to substantially impair molecular transport or diffusion, as numerous FDA‐approved drugs deviate from the conventional Lipinski thresholds of 500 Da for MW and 10 HBA [90]. Overall, although several NPs exhibited Ro5 violations, such deviations are common among bioactive natural compounds and do not necessarily preclude biological activity. Nevertheless, compounds with fewer violations, particularly NPA011203 and NPA012944, emerged as more promising candidates for further optimization, whereas highly noncompliant compounds may require structural modification or alternative delivery strategies.

### 3.5. ADMET Features

Evaluating ADMET properties is essential in early‐stage drug discovery to identify compounds that are not only potent but also pharmacokinetically suitable and safe [91]. Accordingly, the pkCSM ADMET descriptors were determined to evaluate the pharmacokinetic properties of the identified NPAtlas compounds. Notably, the log *S* values for NPA011203, NPA013234, NPA016048, NPA012944, NPA001712, NPA002823, and PBTZ169 were found to be −4.49, −3.00, −2.89, −4.83, −3.98, −2.90, and −5.6 mg/mL, respectively, indicating their reasonable solubility (Table 2). Additionally, the skin permeability (log *Kp*) values of NPA011203, NPA013234, NPA016048, NPA012944, NPA001712, NPA002823, and PBTZ169 were −2.78, −2.74, −2.74, 2.88, −2.73, −2.74, and −2.72, respectively, suggesting their good absorption characteristics (Table 2). Notably, the identified NPAtlas compounds and PBTZ169 exhibited poor BBB permeability, with log BB values ranging from −0.44 to −2.52. Moreover, the log PS permeability values, ranging from −2.1 to −4.66, demonstrated limited CNS penetration, thereby reducing the risk of CNS‐related side effects in anti‐TB agents. Regarding metabolism predictions, both the identified NPAtlas compounds and PBTZ169 were predicted to act as substrates for the CYP3A4 enzyme, with only PBTZ169 identified as an inhibitor. In contrast, NPA011203, NPA013234, NPA016048, NPA012944, NPA001712, and NPA002823 were classified as noninhibitors. All compounds were predicted to be nonsubstrates and noninhibitors for the CYP2D6 enzyme, indicating a minimal risk of drug‐drug interactions (Table 2). Based on excretion properties, the total clearance was determined as 0.52, 0.77, 1.26, 0.52, 0.51, 0.27, and 0.02 mL/min/kg for NPA011203, NPA013234, NPA016048, NPA012944, NPA001712, NPA002823, and PBTZ169, respectively. Toxicity analysis revealed that NPA011203, NPA013234, NPA016048, NPA012944, and NPA002823 were nontoxic, whereas NPA001712 and PBTZ169 were determined to be toxic. No hERG I inhibition was observed, indicating low cardiotoxic risk; however, PBTZ169, NPA013234, NPA001712, and NPA002823 were predicted to inhibit hERG II (Table 2). The estimated oral acute toxicity (LD50) values varied from 2.34 to 2.63 mol/kg, reflecting moderate toxicity. Hepatotoxicity was forecasted for PBTZ169, NPA011203, NPA013234, and NPA016048, while NPA012944, NPA001712, and NPA002823 were anticipated to be non‐hepatotoxic (Table 2). Importantly, the skin sensitivity test confirmed the absence of reactivity for the identified NPAtlas compounds and PBTZ169, underscoring the safety of these compounds upon contact. Taken together, the identified NPAtlas compounds produced acceptable ADMET results; some of the metrics were higher than those of PBTZ169.

**TABLE 2:** The estimated ADMET profile of the identified NPAtlas compounds and PBZ169.

ADMET features		PBTZ169	NPA011203	NPA013234	NPA016048	NPA012944	NPA001712	NPA002823
Absorption	Water solubility (log *S*)	−5.6	−4.49	−3.00	−2.89	−4.83	−3.98	−2.90
	Skin permeability (log *Kp*)	−2.72	−2.78	−2.74	−2.74	−2.88	−2.73	−2.74

Distribution	Blood–brain barrier (BBB)	−0.90	−0.85	−1.73	−2.09	−0.44	−2.03	−2.52
	CNS permeability (log PS)	−2.10	−2.57	−4.11	−3.90	−2.73	−2.72	−4.66

Metabolism	CYP3A4 substrate	Yes	Yes	Yes	Yes	Yes	Yes	Yes
	CYP3A4 inhibitor	Yes	No	No	No	No	No	No
	CYP2D6 substrate	No	No	No	No	No	No	No
	CYP2D6 inhibitor	No	No	No	No	No	No	No

Excretion	Total clearance	0.02	0.52	0.77	1.26	0.52	0.51	0.27

Toxicity	AMES toxicity	Yes	No	No	No	No	Yes	No
	hERG I inhibitor	No	No	No	No	No	No	No
	hERG II inhibitor	Yes	No	Yes	No	No	Yes	Yes
	Oral rat acute toxicity (LD50)	2.63	2.38	2.39	2.48	2.34	2.38	2.62
	Hepatotoxicity	Yes	Yes	Yes	Yes	No	No	No
	Skin sensitization	No	No	No	No	No	No	No

## 4. Conclusion

The current research used an integrated irreversible covalent docking and MDS to determine the potentiality of 133 nitro‐containing NPAtlas compounds as DprE1 inhibitors, accompanied by MM‐GBSA binding energy estimations. *In-silico* MM‐GBSA//250 ns MDS computations suggested that the identified six NPAtlas compounds possessed superior binding affinities to DprE1 compared to PBTZ169 (Δ*G*_binding_ = −49.4 kcal·mol^−1^). The identified NPAtlas compounds demonstrated advantageous energetic and structural traits, along with encouraging drug‐like properties and oral BA. The current study suggested that the identified NPAtlas compounds are potentially promising inhibitors of DprE1, justifying further evaluation in in vitro/in vivo studies.

## Author Contributions

Conceptualization: Mahmoud A. A. Ibrahim, and Yanshuo Han; methodology: Mahmoud A. A. Ibrahim; software: Mahmoud A. A. Ibrahim; formal analysis: Doaa G. M. Mahmoud; investigation: Doaa G. M. Mahmoud; resources: Mahmoud A. A. Ibrahim and Tarad Abalkhail; data curation: Doaa G. M. Mahmoud; writing–original draft preparation: Doaa G. M. Mahmoud; visualization: Doaa G. M. Mahmoud, Peter A. Sidhom, and Tarad Abalkhail; supervision: Mahmoud A. A. Ibrahim, Gamal A. H. Mekhemer, and Mohamed‐Elamir F. Hegazy.; and project administration: Mahmoud A. A. Ibrahim, Sherif S. Ebada, and Yanshuo Han; writing–review and editing: Mahmoud A. A. Ibrahim; Sherif S. Ebada, Peter A. Sidhom, Gamal A. H. Mekhemer, Mohamed‐Elamir F. Hegazy, Yanshuo Han, and Tarad Abalkhail.

## Funding

This research was funded by Ongoing Research Funding Program (ORF‐2026‐1438), King Saud University, Riyadh, Saudi Arabia.

## Disclosure

All authors have read and agreed to the published version of the manuscript.

## Consent

The authors have nothing to report.

## Conflicts of Interest

The authors declare no conflicts of interest.

## Supporting Information

Figure S1: Flow diagram outlining the steps involved in NPAtlas compound preparation; Figure S2: 2D representations of the predicted binding modes of the most probable NPAtlas compounds complexed with DprE1; Figure S3: 2D representations of the predicted binding modes for (a) NPA011203, (b) NPA013234, (c) NPA016048, (d) NPA012944, (e) NPA001712, (f) NPA002823, and (g) PBTZ169 with DprE1, generated from representative structures extracted at 50 ns MDS; Figure S4: 2D representations of the predicted binding modes for (a) NPA011203, (b) NPA013234, (c) NPA016048, (d) NPA012944, (e) NPA001712, (f) NPA002823, and (g) PBTZ169 with DprE1, generated from representative structures extracted at 100 ns; Figure S5: 2D representations of the predicted binding modes for (a) NPA011203, (b) NPA013234, (c) NPA016048, (d) NPA012944, (e) NPA001712, (f) NPA002823, and (g) PBTZ169 with DprE1, generated from representative structures extracted at 150 ns MDS; Figure S6: 2D representations of the predicted binding modes for (a) NPA011203, (b) NPA013234, (c) NPA016048, (d) NPA012944, (e) NPA001712, (f) NPA002823, and (g) PBTZ169 with DprE1, generated from representative structures extracted at 200 ns MDS; Table S1: Calculated covalent docking scores (in kcal·mol^−1^) of 133 NPAtlas compounds and PBTZ169 against DprE1; Table S2: Estimated covalent docking scores and MM‐GBSA binding energies (in kcal·mol^−1^) over 5 ns MDS of the top 47 NPAtlas compounds and PBTZ169 against DprE1; Table S3: Computed covalent docking scores and MM‐GBSA binding energies (in kcal·mol^−1^) over 5 and 25 ns MDS of the top 19 NPAtlas compounds against DprE1; Table S4: Estimated covalent docking scores and MM‐GBSA binding energies (in kcal·mol^−1^) over 5, 25, and 100 ns MDS of the top 11 NPAtlas compounds and PBTZ169 against DprE1; Table S5: Estimated number of the rotatable bond (RB), synthetic accessibility (SA) score, and bioavailability (BA) score of the identified NPAtlas compounds and PBZ169.

## Supporting information


**Supporting Information** Additional supporting information can be found online in the Supporting Information section.

## Data Availability

The data that support the findings of this study are available in the supporting information of this article.
