# Supplementary material for: Exploring the Natural Products Atlas (NPAtlas) Database for Hunting Prospective Irreversible Covalent DprE1 Inhibitors With Antitubercular Activity: An Integrated In-Silico Approach
Source: J Trop Med. 2026 Feb 14;2026:8879019. doi: 10.1155/jotm/8879019 (PMC12906241; doi:10.1155/jotm/8879019)
Supplement: Supplementary file 1 — Supporting Information Additional supporting information can be found online in the Supporting Information section. [file JOTM-2026-8879019-s001.docx]

**
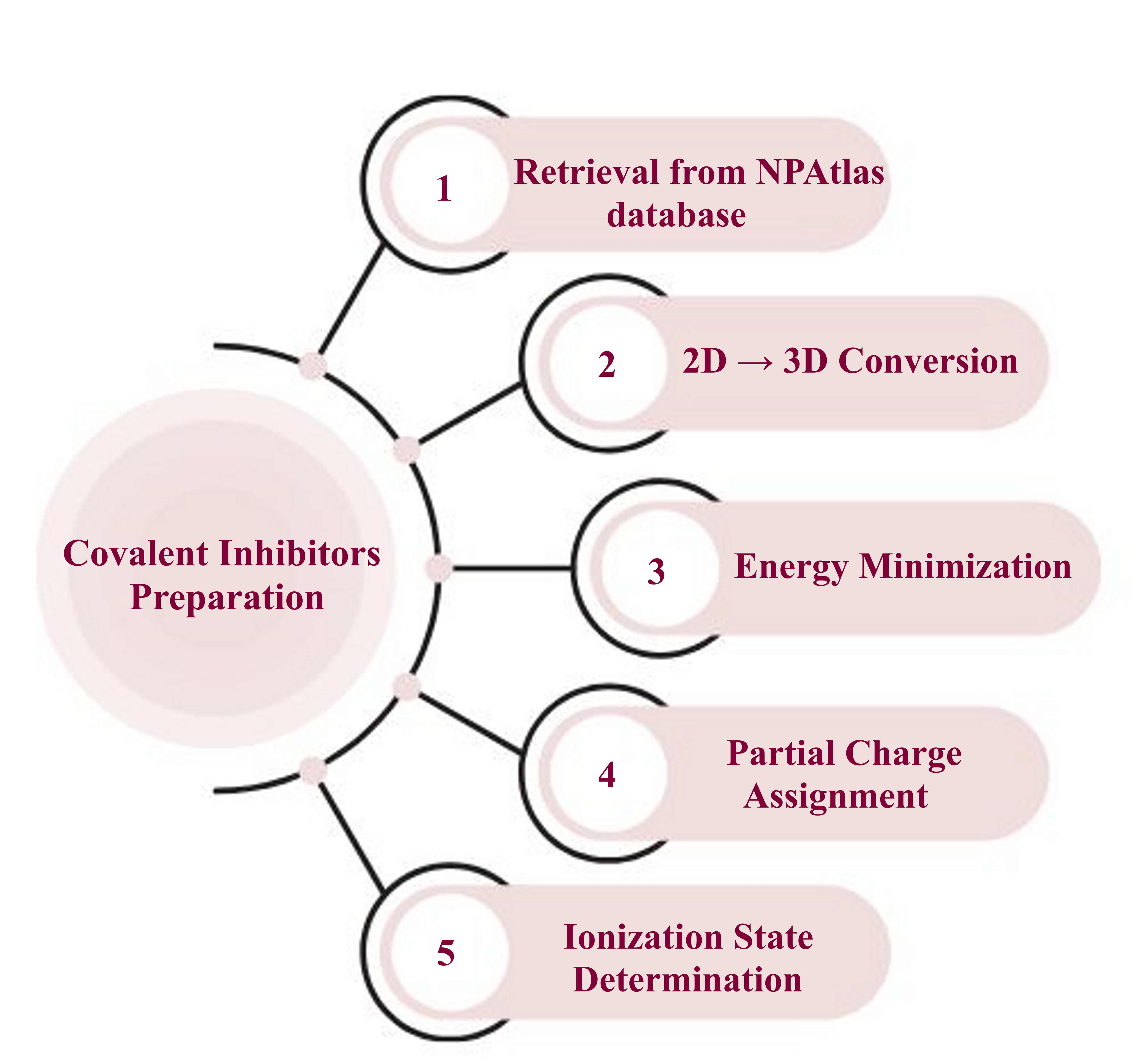
**

**Figure S1.** Flow diagram outlining the steps involved in NPAtlas compound preparation.

**
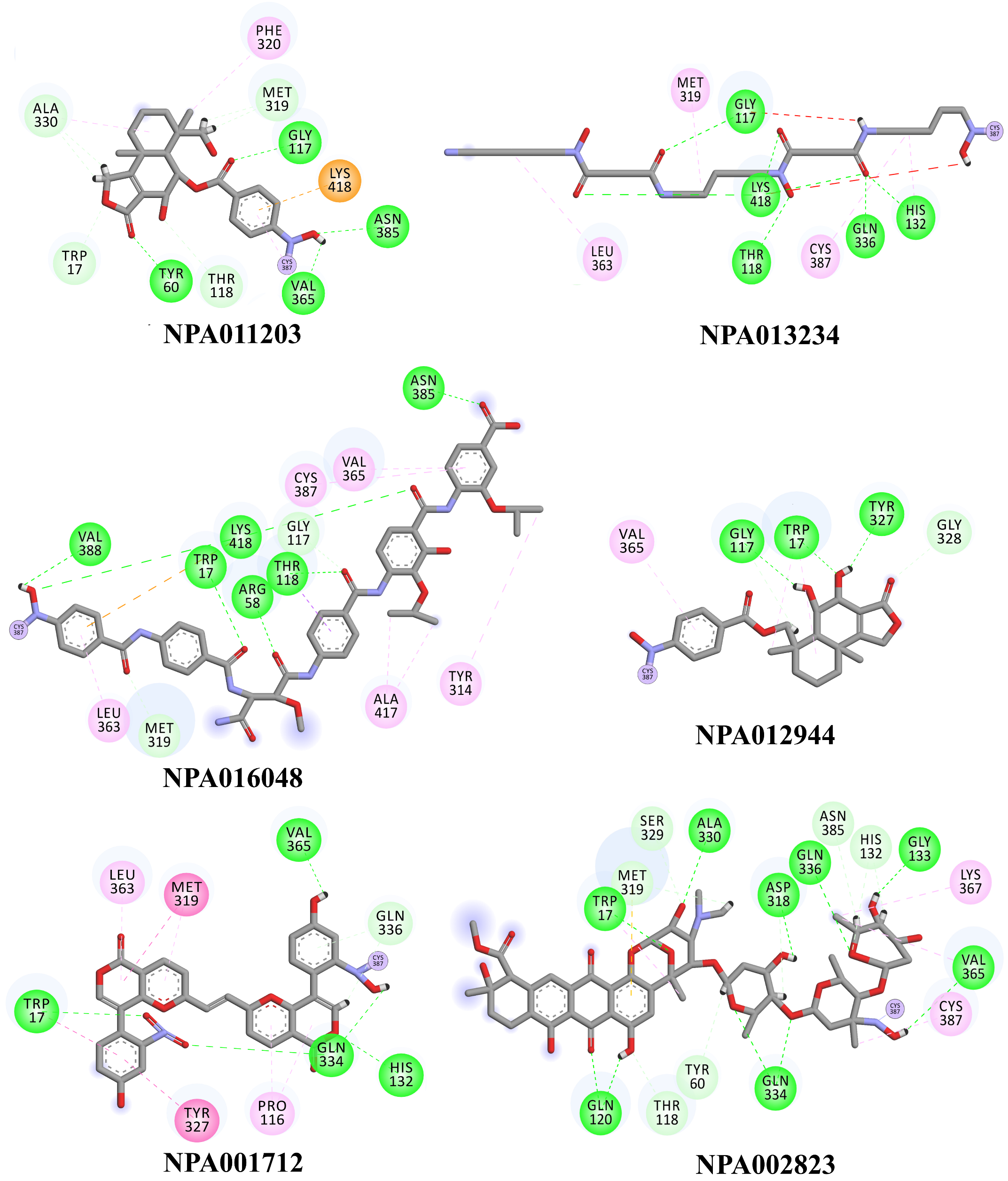
**

**Figure S2.** 2D representations of the predicted binding modes of the most potent NPAtlas compounds complexed with DprE1.

**
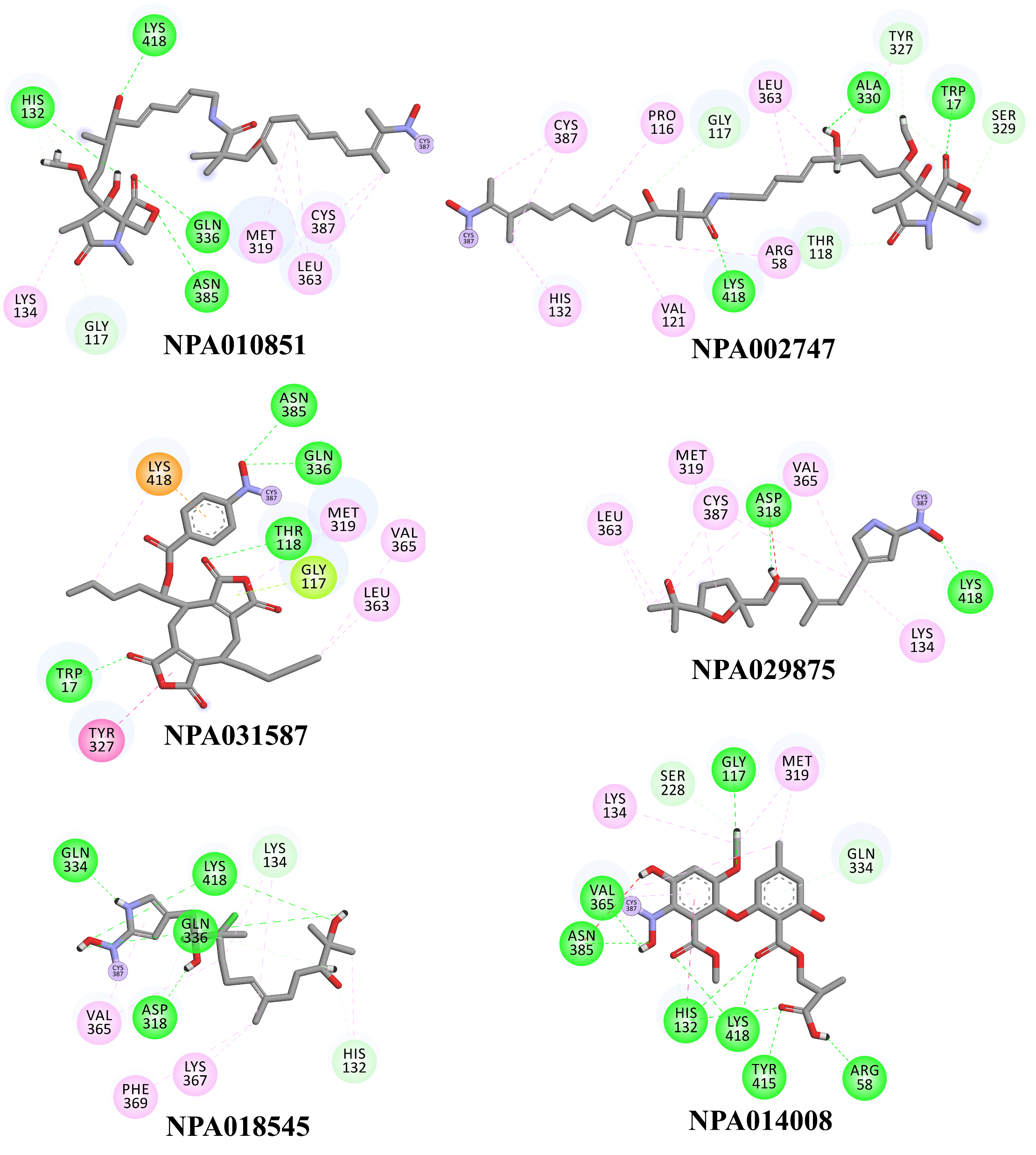
**

**Figure S2.** *Continued*.

**
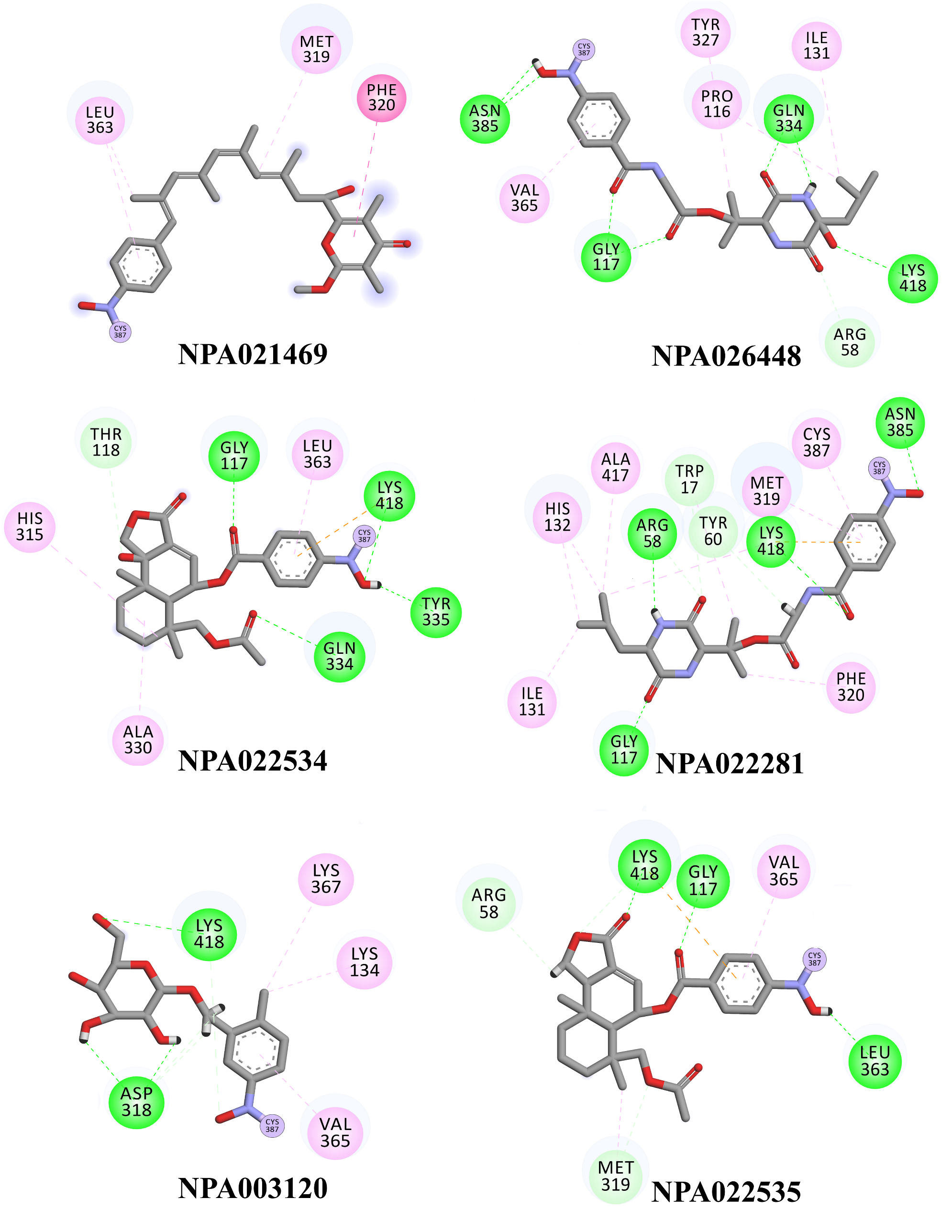
**

**Figure S2.** *Continued*.

**
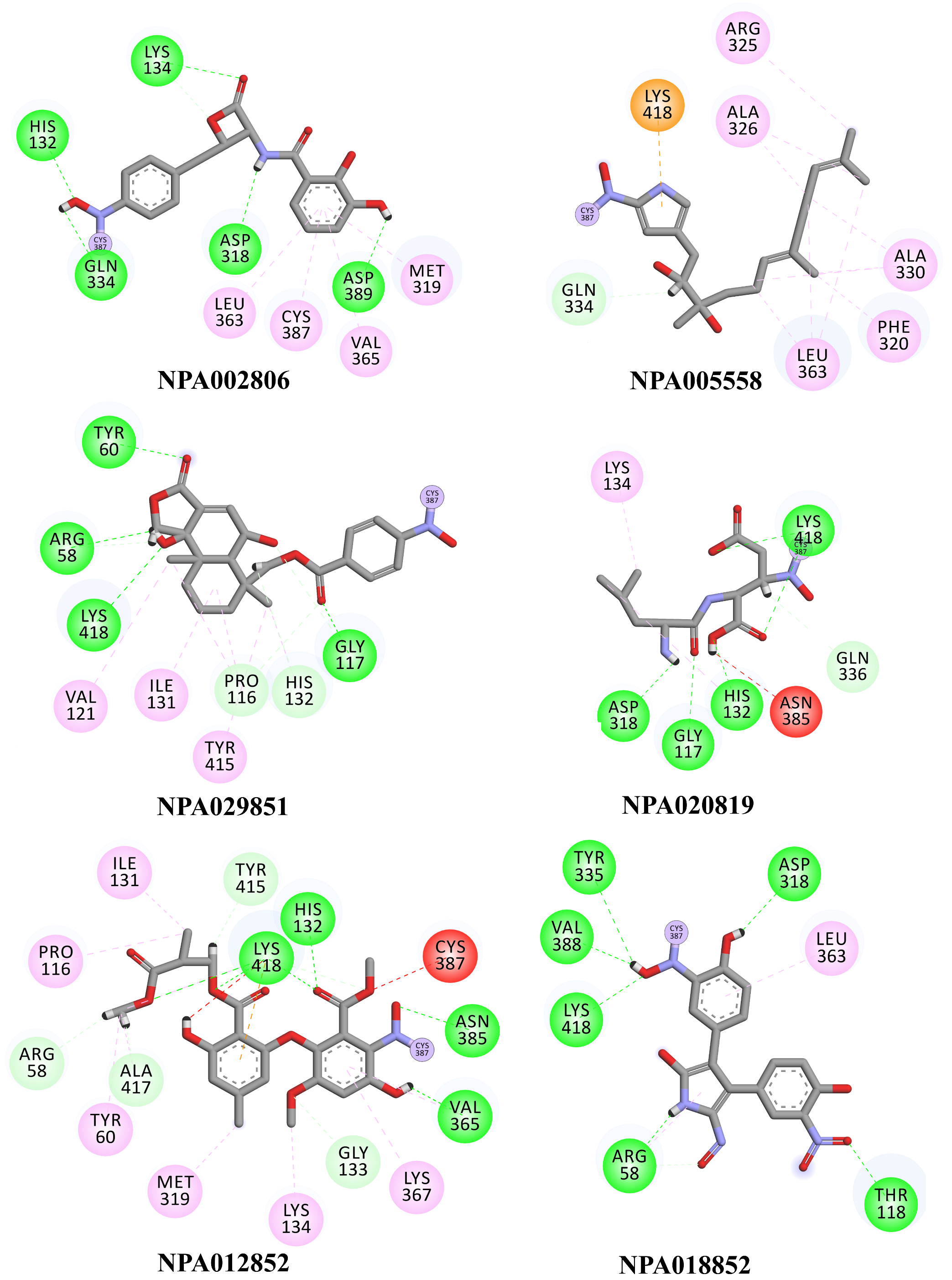
**

**Figure S2.** *Continued*.


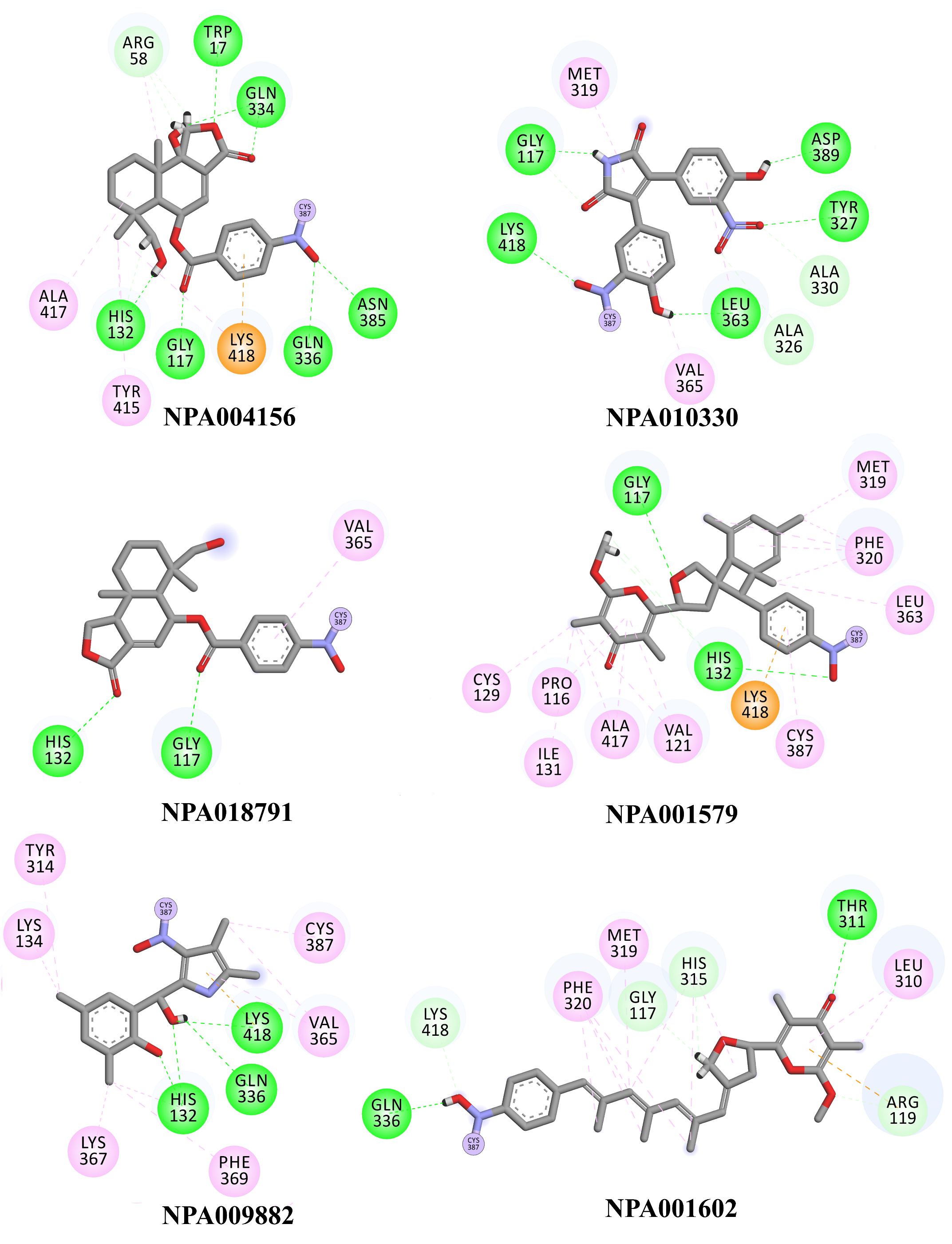


**Figure S2.** *Continued*.

**
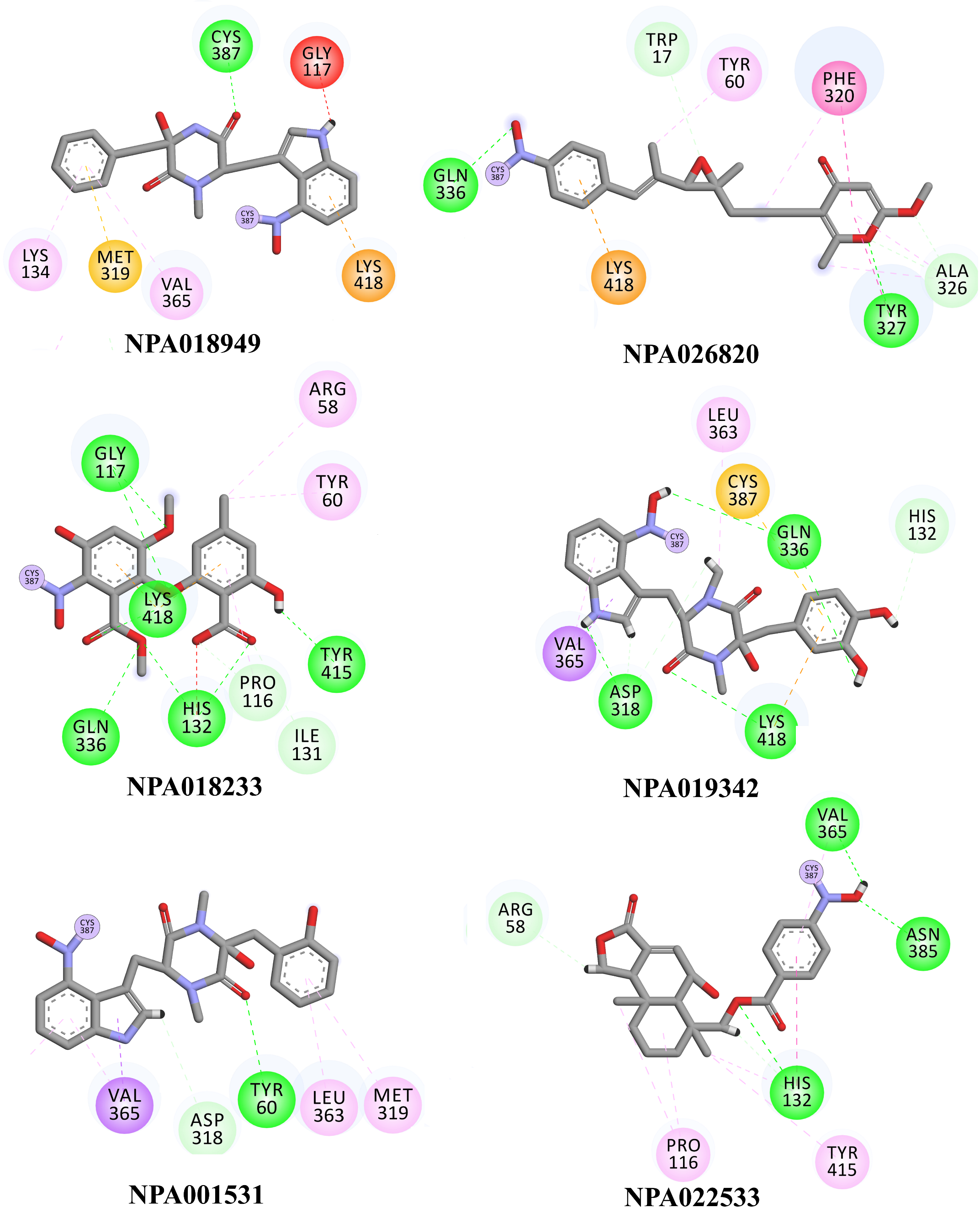
**

**Figure S2.** *Continued*.

**
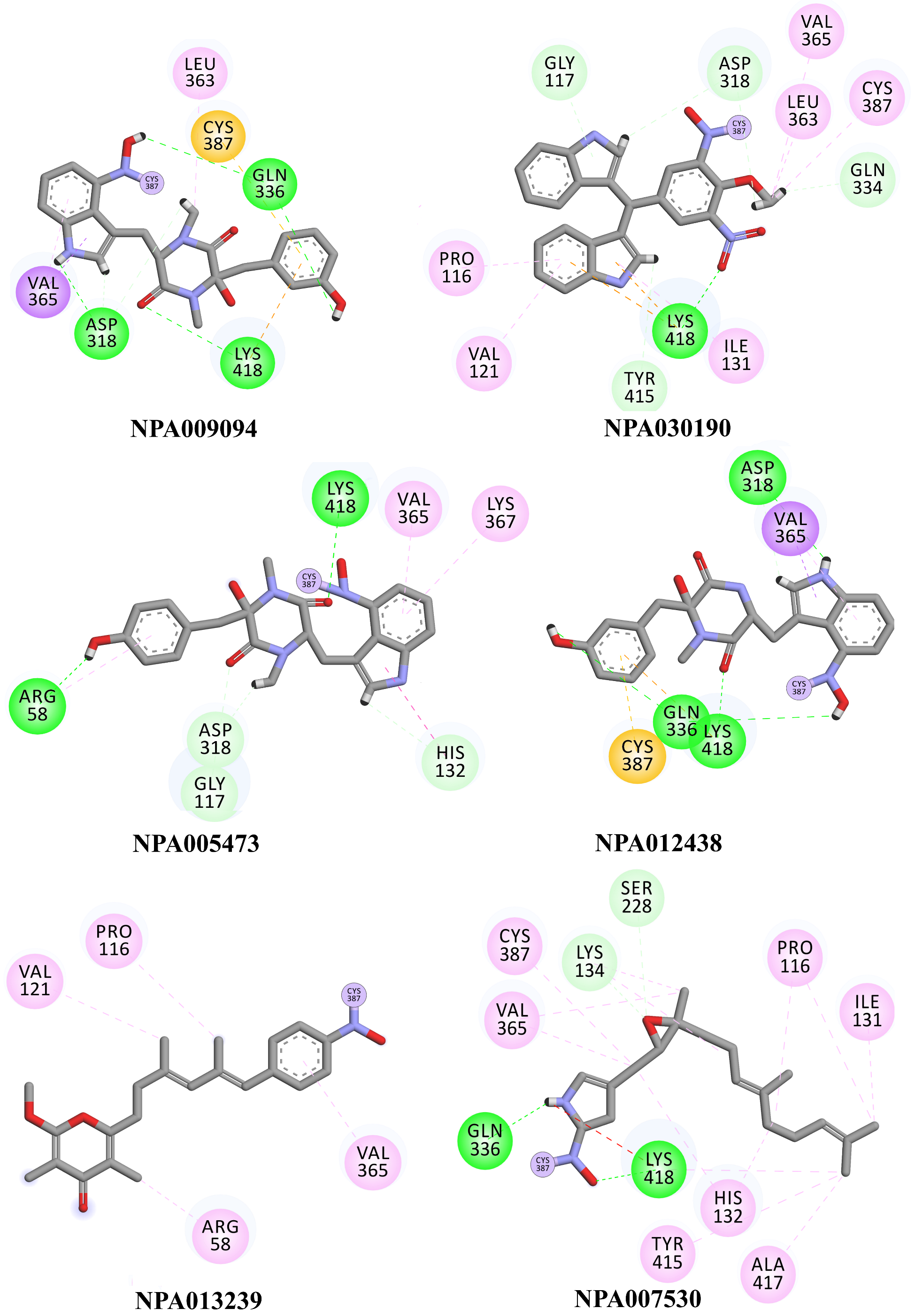
**

**Figure S2.** *Continued*.

**
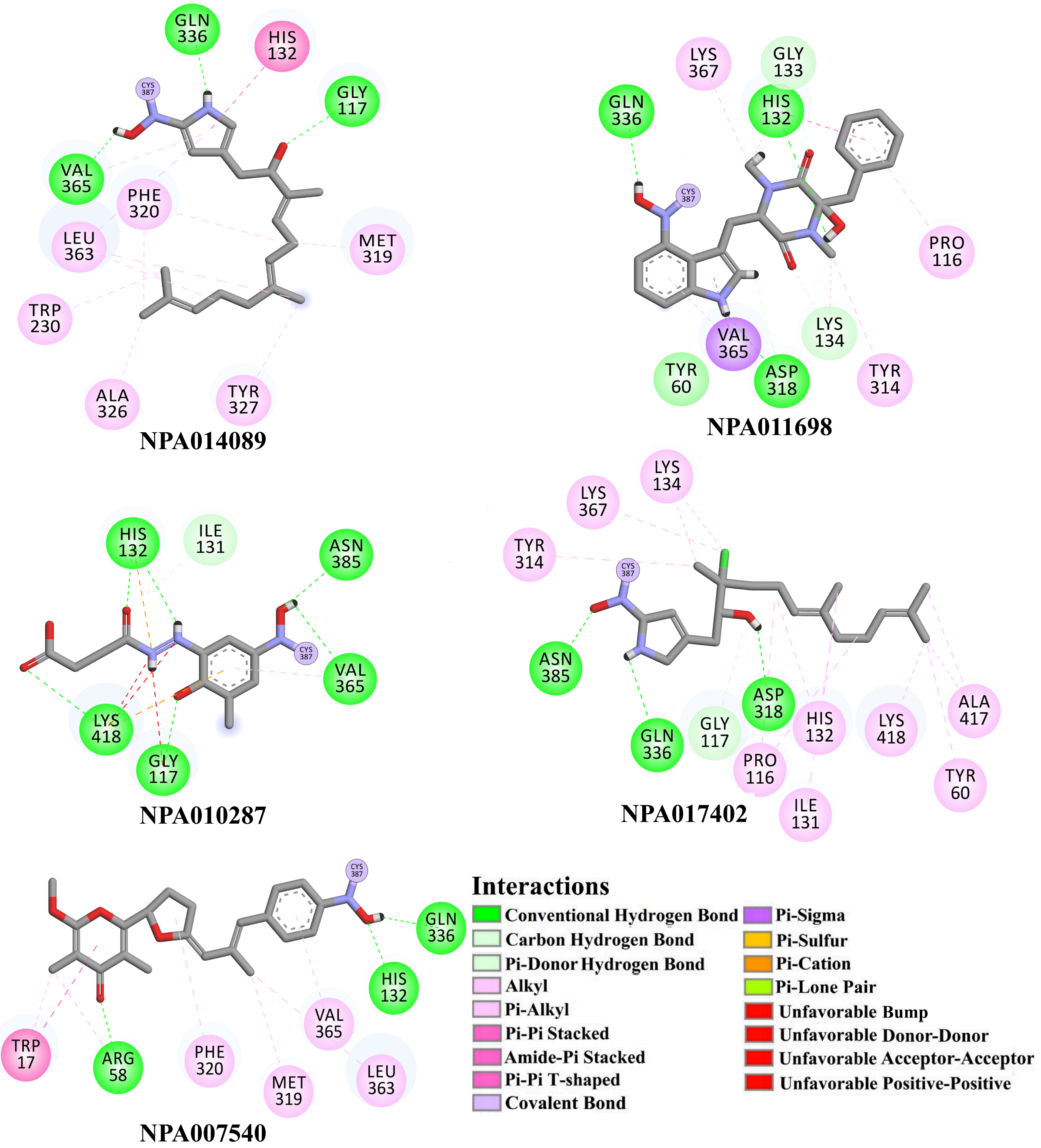
**

**Figure S2.** *Continued*.


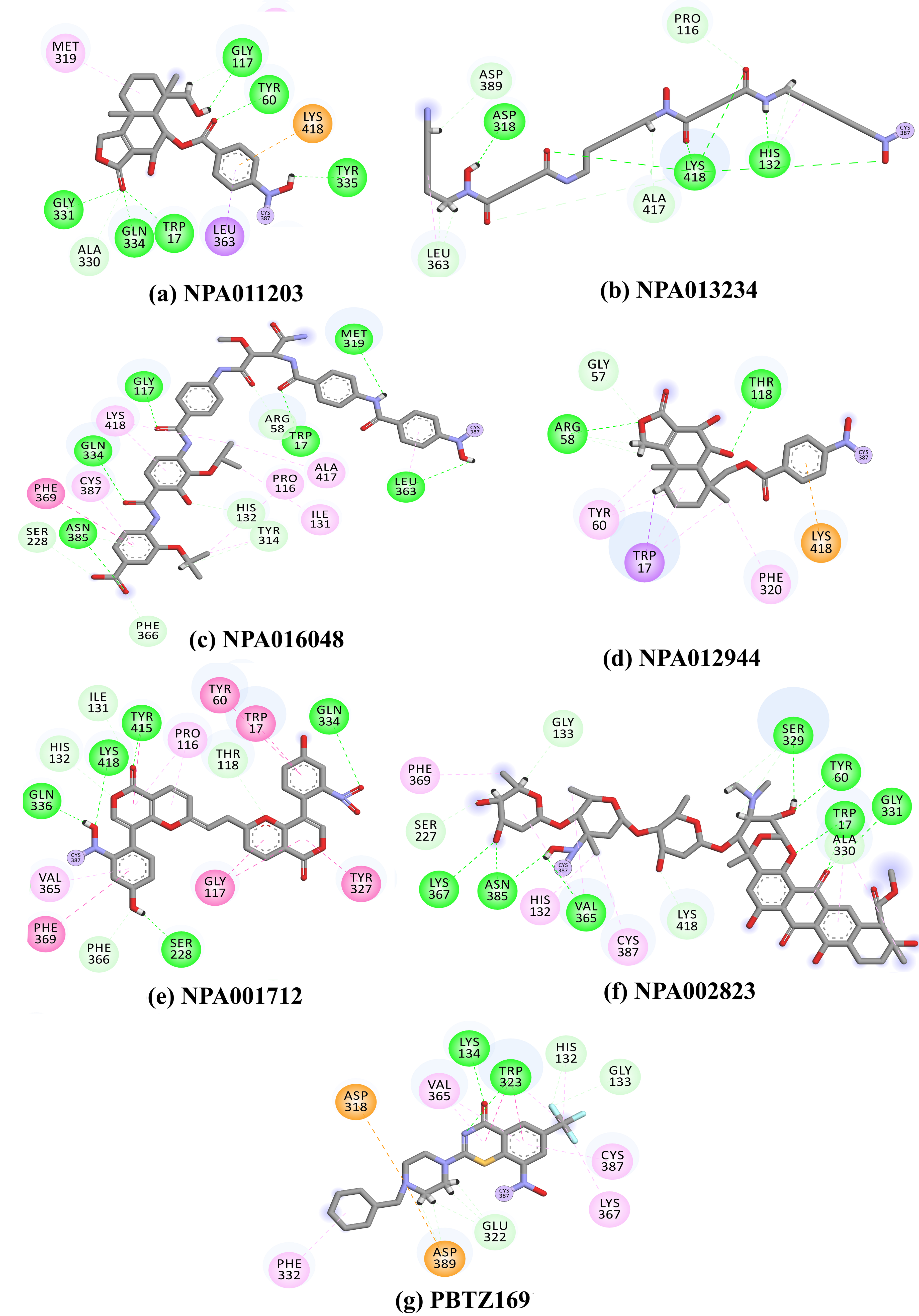


**Figure S3**. 2D represntations of the predicted binding modes for (a) NPA011203, (b) NPA013234, (c) NPA016048, (d) NPA012944, (e) NPA001712, (f) NPA002823, and (g) PBTZ169 with DprE1, generated from representative structures extracted at 50 ns MDS.


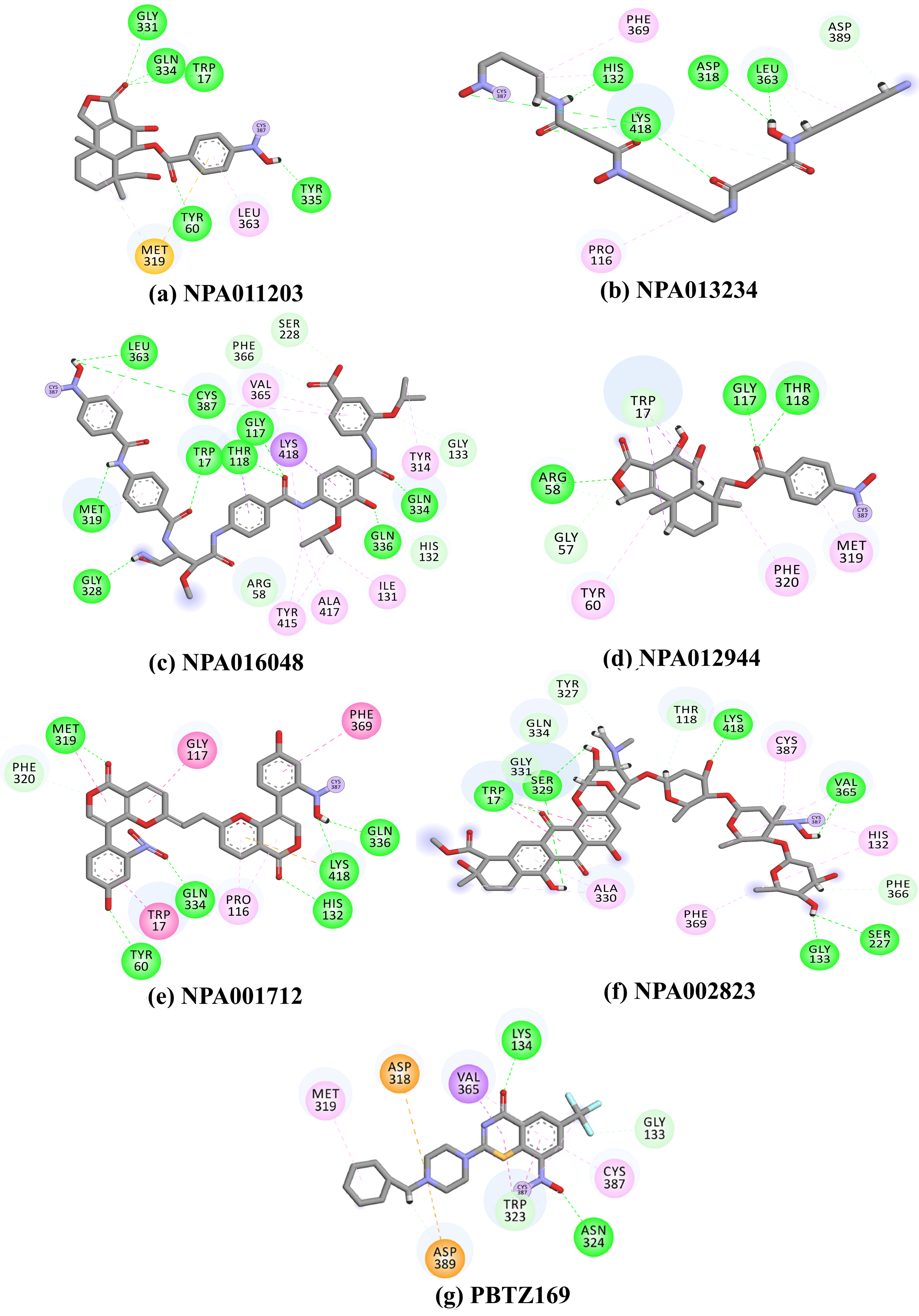


**Figure S4**. 2D representations of the predicted binding modes for (a) NPA011203, (b) NPA013234, (c) NPA016048, (d) NPA012944, (e) NPA001712, (f) NPA002823, and (g) PBTZ169 with DprE1, generated from representative structures extracted at 100 ns.


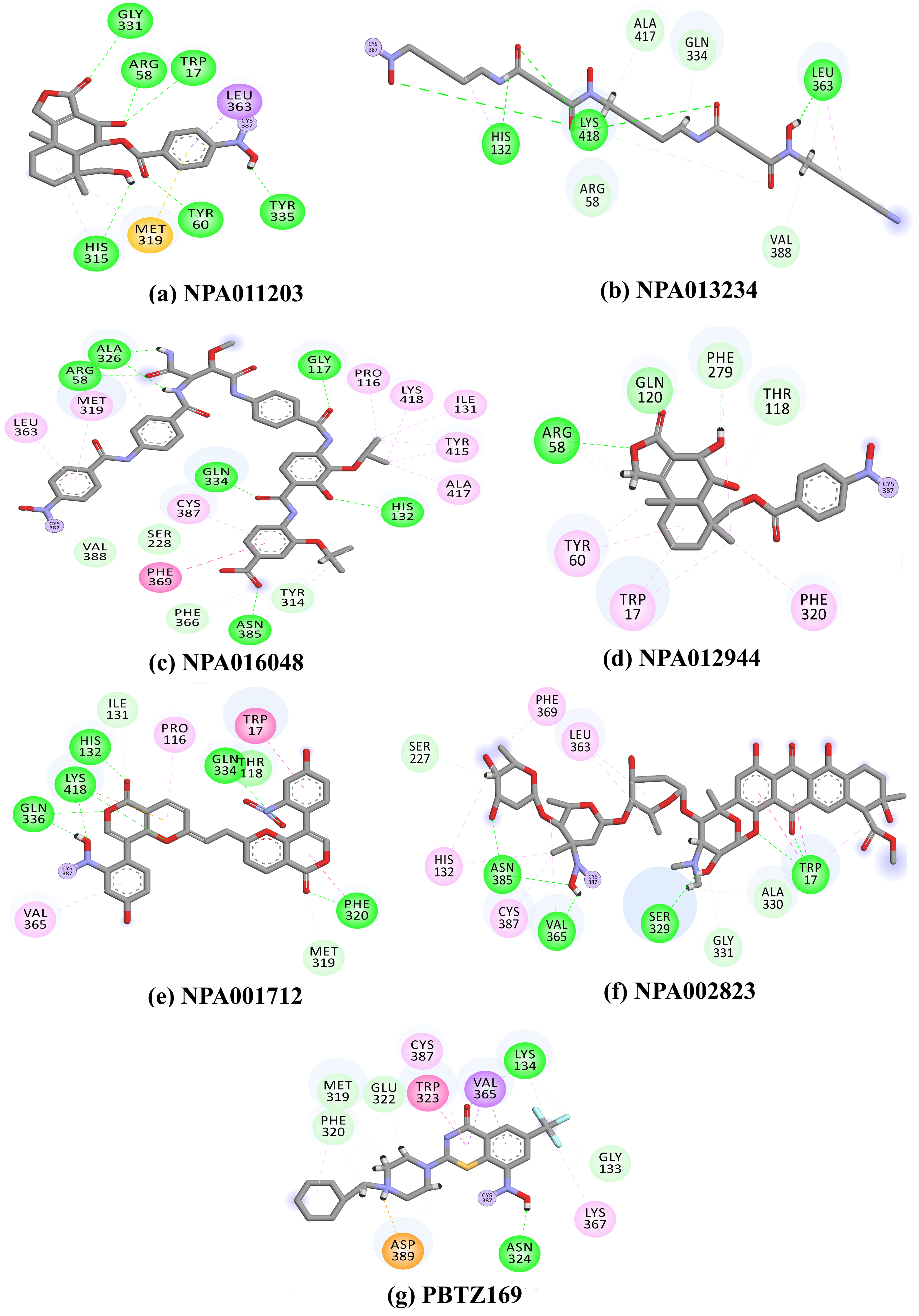


**Figure S5**. 2D represntations of the predicted binding modes for (a) NPA011203, (b) NPA013234, (c) NPA016048, (d) NPA012944, (e) NPA001712, (f) NPA002823, and (g) PBTZ169 with DprE1, generated from representative structures extracted at 150 ns MDS.


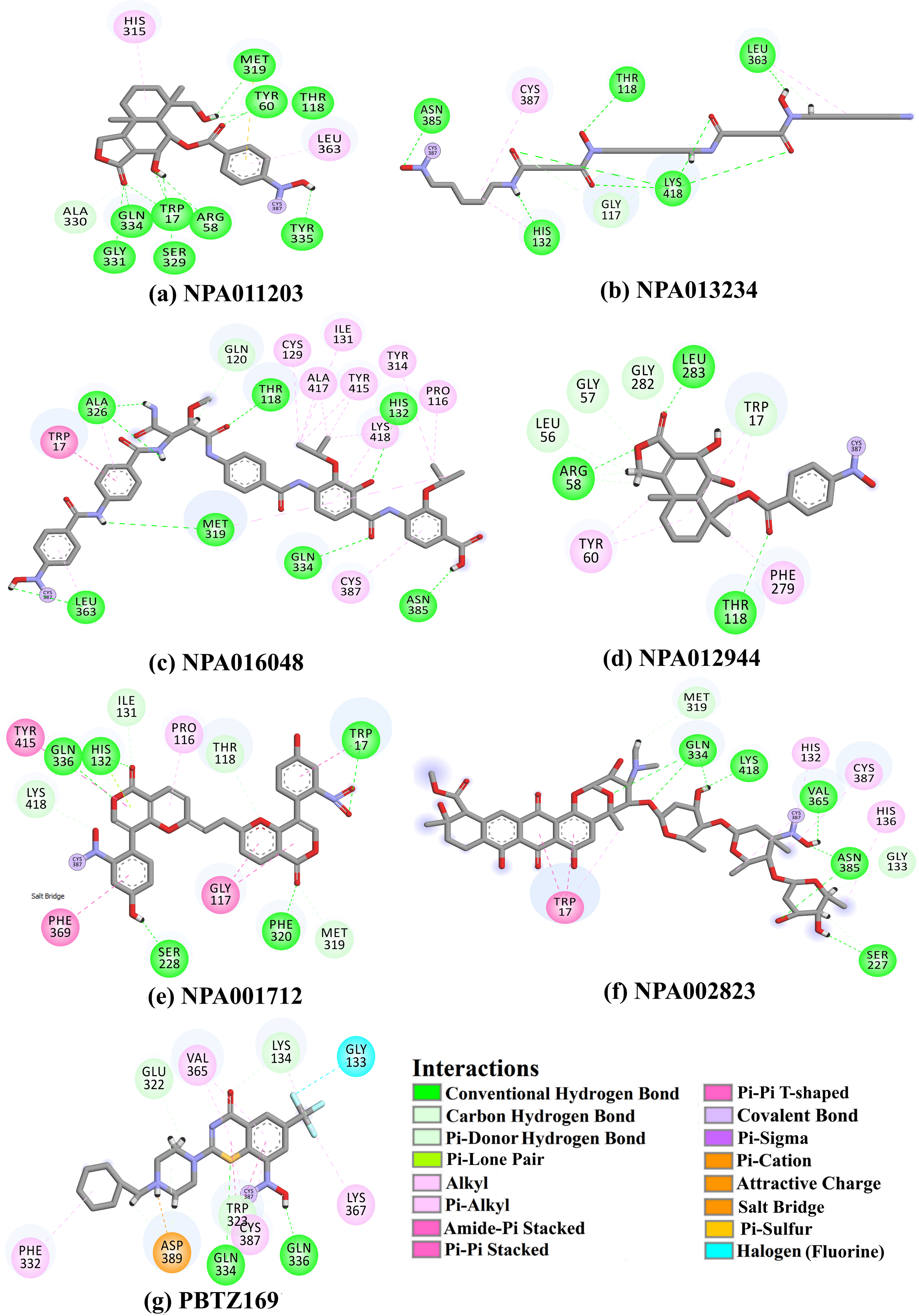


**Figure S6**. 2D representations of the predicted binding modes for (a) NPA011203, (b) NPA013234, (c) NPA016048, (d) NPA012944, (e) NPA001712, (f) NPA002823, and (g) PBTZ169 with DprE1, generated from representative structures extracted at 200 ns MDS.

### Table S1. Calculated covalent docking scores (in kcal.mol^−1^) of 133 NPAtlas compounds and PBTZ169 against DprE1.

| **No.** | **NPAtlas Code** | **Covalent Docking Score (kcal.mol^−1^)** | **No.** | **NPAtlas Code** | **Covalent Docking Score (kcal.mol^−1^)** |
| --- | --- | --- | --- | --- | --- |
|  | PBTZ169 (Macozinone) | –7.8 | 44 | NPA011698 | –8.0 |
| 1 | NPA011203 | –15.8 | 45 | NPA010287 | –7.9 |
| 2 | NPA013234 | –15.3 | 46 | NPA017402 | –7.9 |
| 3 | NPA016048 | –15.1 | 47 | NPA007540 | –7.9 |
| 4 | NPA012944 | –14.7 | 48 | NPA003643 | –7.7 |
| 5 | NPA001712 | –14.4 | 49 | NPA007238 | –7.7 |
| 6 | NPA002823 | –13.9 | 50 | NPA016200 | –7.7 |
| 7 | NPA010851 | –13.8 | 51 | NPA004794 | –7.5 |
| 8 | NPA002747 | –13.2 | 52 | NPA016382 | –7.5 |
| 9 | NPA031587 | –11.5 | 53 | NPA017817 | –7.4 |
| 10 | NPA029875 | –10.4 | 54 | NPA003790 | –7.4 |
| 11 | NPA018545 | –10.4 | 55 | NPA022030 | –7.3 |
| 12 | NPA014008 | –10.1 | 56 | NPA033050 | –7.3 |
| 13 | NPA021469 | –10.1 | 57 | NPA020410 | –7.3 |
| 14 | NPA026448 | –10.0 | 58 | NPA000004 | –7.3 |
| 15 | NPA022534 | –9.7 | 59 | NPA013432 | –7.2 |
| 16 | NPA022281 | –9.5 | 60 | NPA017302 | –7.2 |
| 17 | NPA003120 | –9.5 | 61 | NPA033051 | –7.1 |
| 18 | NPA022535 | –9.4 | 62 | NPA018670 | –7.0 |
| 19 | NPA002806 | –9.4 | 63 | NPA032756 | –6.9 |
| 20 | NPA005558 | –9.2 | 64 | NPA020405 | –6.9 |
| 21 | NPA029851 | –9.1 | 65 | NPA015582 | –6.8 |
| 22 | NPA020819 | –9.1 | 66 | NPA014098 | –6.7 |
| 23 | NPA012852 | –9.0 | 67 | NPA018409 | –6.7 |
| 24 | NPA018852 | –9.0 | 68 | NPA028530 | –6.6 |
| 25 | NPA004156 | –8.8 | 69 | NPA004980 | –6.5 |
| 26 | NPA010330 | –8.7 | 70 | NPA016248 | –6.5 |
| 27 | NPA018791 | –8.7 | 71 | NPA011246 | –6.5 |
| 28 | NPA001579 | –8.6 | 72 | NPA016373 | –6.5 |
| 29 | NPA009882 | –8.6 | 73 | NPA016700 | –6.4 |
| 30 | NPA001602 | –8.6 | 74 | NPA004481 | –6.2 |
| 31 | NPA018949 | –8.5 | 75 | NPA028532 | –6.2 |
| 32 | NPA026820 | –8.4 | 76 | NPA005160 | –6.1 |
| 33 | NPA018233 | –8.4 | 77 | NPA008691 | –5.9 |
| 34 | NPA019342 | –8.4 | 78 | NPA017599 | –5.9 |
| 35 | NPA001531 | –8.3 | 79 | NPA005355 | –5.8 |
| 36 | NPA022533 | –8.3 | 80 | NPA009468 | –5.7 |
| 37 | NPA009094 | –8.2 | 81 | NPA003805 | –5.7 |
| 38 | NPA030190 | –8.2 | 82 | NPA020813 | –5.6 |
| 39 | NPA005473 | –8.2 | 83 | NPA019605 | –5.6 |
| 40 | NPA012438 | –8.2 | 84 | NPA007994 | –5.6 |
| 41 | NPA013239 | –8.1 | 85 | NPA007990 | –5.4 |
| 42 | NPA007530 | –8.1 | 86 | NPA026193 | –5.3 |
| 43 | NPA014089 | –8.0 | 87 | NPA022029 | –5.3 |

### Table S1. *Continued*.

| **No.** | **NPAtlas Code** | **Covalent Docking Score (kcal.mol^−1^)** | **No.** | **NPAtlas Code** | **Covalent Docking Score (kcal.mol^−1^)** |
| --- | --- | --- | --- | --- | --- |
| 88 | NPA011608 | –5.3 | 111 | NPA006605 | –3.9 |
| 89 | NPA009873 | –5.2 | 112 | NPA016081 | –3.7 |
| 90 | NPA004596 | –5.2 | 113 | NPA010125 | –3.7 |
| 91 | NPA020639 | –5.1 | 114 | NPA005775 | –3.6 |
| 92 | NPA029208 | –5.1 | 115 | NPA009720 | –3.4 |
| 93 | NPA001283 | –4.9 | 116 | NPA015230 | –3.3 |
| 94 | NPA027885 | –4.9 | 117 | NPA016461 | –3.2 |
| 95 | NPA027869 | –4.8 | 118 | NPA013732 | –3.0 |
| 96 | NPA011131 | –4.6 | 119 | NPA028529 | –2.9 |
| 97 | NPA020960 | –4.6 | 120 | NPA014508 | –2.9 |
| 98 | NPA027884 | –4.6 | 121 | NPA006950 | –2.8 |
| 99 | NPA029849 | –4.5 | 122 | NPA011158 | –2.8 |
| 100 | NPA006203 | –4.5 | 123 | NPA020404 | –2.8 |
| 101 | NPA018752 | –4.4 | 124 | NPA007876 | –2.8 |
| 102 | NPA020929 | –4.4 | 125 | NPA005686 | –2.7 |
| 103 | NPA013917 | –4.4 | 126 | NPA019603 | –2.2 |
| 104 | NPA006255 | –4.4 | 127 | NPA028531 | –1.8 |
| 104 | NPA011560 | –4.3 | 128 | NPA029946 | –1.8 |
| 106 | NPA017839 | –4.3 | 129 | NPA018700 | –1.5 |
| 107 | NPA005622 | –4.2 | 130 | NPA022582 | –1.2 |
| 108 | NPA028528 | –4.1 | 131 | NPA030411 | –1.1 |
| 109 | NPA006531 | –3.9 | 132 | NPA022583 | –1.0 |
| 110 | NPA008941 | –3.9 | 133 | NPA006547 | –0.6 |

**Table S2.** Estimated covalent docking scores and MM-GBSA binding energies (in kcal.mol^−1^) over 5 ns MDS of the top 47 NPAtlas compounds and PBTZ169 against DprE1 ^a^.

| **No.** | **NPAtlas**  **Code** | **Covalent Docking Score** | **MM-GBSA Binding Energy** | **No.** | **NPAtlas Code** | **Covalent Docking Score** | **MM-GBSA Binding Energy** |
| --- | --- | --- | --- | --- | --- | --- | --- |
|  | PBTZ169 (Macozinone) | –7.8 | –37.9 | 24 | NPA002747 | –13.2 | –35.1 |
| 1 | NPA011203 | –15.8 | –75.7 | 25 | NPA012438 | –8.2 | –34.9 |
| 2 | NPA013234 | –15.3 | –68.6 | 26 | NPA001531 | –8.3 | –34.9 |
| 3 | NPA016048 | –15.1 | –62.2 | 27 | NPA005558 | –9.2 | –33.9 |
| 4 | NPA014008 | –10.1 | –57.1 | 28 | NPA026820 | –8.4 | –33.3 |
| 5 | NPA002823 | –13.9 | –56.9 | 29 | NPA003120 | –9.5 | –33.2 |
| 6 | NPA012944 | –14.7 | –56.1 | 30 | NPA022535 | –9.4 | –33.0 |
| 7 | NPA001712 | –14.4 | –49.4 | 31 | NPA018949 | –8.5 | –32.8 |
| 8 | NPA029875 | –10.4 | –47.3 | 32 | NPA026448 | –10.0 | –32.2 |
| 9 | NPA010851 | –13.8 | –46.2 | 33 | NPA021469 | –10.1 | –31.0 |
| 10 | NPA005473 | –8.2 | –43.2 | 34 | NPA022281 | –9.5 | –30.0 |
| 11 | NPA030190 | –8.2 | –43.1 | 35 | NPA009882 | –8.6 | –29.1 |
| 12 | NPA007540 | –7.8 | –42.7 | 36 | NPA018233 | –8.4 | –28.8 |
| 13 | NPA002806 | –9.4 | –42.6 | 37 | NPA013239 | –8.1 | –28.3 |
| 14 | NPA009094 | –8.2 | –41.5 | 38 | NPA010330 | –8.7 | –26.3 |
| 15 | NPA012852 | –9.0 | –41.2 | 39 | NPA018852 | –9.0 | –25.5 |
| 16 | NPA007530 | –8.1 | –40.4 | 40 | NPA022533 | –8.3 | –24.1 |
| 17 | NPA018545 | –10.4 | –40.3 | 41 | NPA018791 | –8.7 | –22.2 |
| 18 | NPA014089 | –8.0 | –39.8 | 42 | NPA029851 | –9.1 | –21.6 |
| 19 | NPA019342 | –8.4 | –38.5 | 43 | NPA004156 | –8.8 | –17.8 |
| 20 | NPA011698 | –8.0 | –37.4 | 44 | NPA010287 | –7.9 | –17.7 |
| 21 | NPA031587 | –11.5 | –37.0 | 45 | NPA020819 | –9.1 | –16.8 |
| 22 | NPA017402 | –7.9 | –36.6 | 46 | NPA001579 | –8.6 | –16.4 |
| 23 | NPA001602 | –8.6 | –36.1 | 47 | NPA022534 | –9.7 | –9.8 |

^a^ Data ranked based on the MM-GBSA binding energy over the 5 ns MDS.

**Table S3.** Computed covalent docking scores and MM-GBSA binding energies (in kcal.mol^−1^) over 5 and 25 ns MDS of the top 19 NPAtlas compounds against DprE1 ^a^.

| **No.** | **NPAtlas Code** | **Covalent Docking Score (kcal.mol^−1^)** | **MM-GBSA Binding Energy (kcal.mol^−1^)** | |
| --- | --- | --- | --- | --- |
|  |  |  | **5 ns** | **25 ns** |
|  | PBTZ169 (Macozinone) | –7.8 | –37.9 | –42.9 |
| 1 | NPA011203 | –15.8 | –75.7 | –73.3 |
| 2 | NPA013234 | –15.3 | –68.6 | –63.3 |
| 3 | NPA016048 | –15.1 | –62.2 | –62.0 |
| 4 | NPA012944 | –14.7 | –56.1 | –52.8 |
| 5 | NPA002823 | –13.9 | –56.9 | –52.7 |
| 6 | NPA001712 | –14.4 | –49.4 | –52.4 |
| 7 | NPA010851 | –13.8 | –46.2 | –47.8 |
| 8 | NPA014008 | –10.1 | –57.1 | –46.1 |
| 9 | NPA030190 | –8.2 | –43.1 | –45.1 |
| 10 | NPA029875 | –10.4 | –47.3 | –43.8 |
| 11 | NPA007530 | –8.1 | –40.4 | –43.0 |
| 12 | NPA005473 | –8.2 | –43.2 | –42.2 |
| 13 | NPA009094 | –8.2 | –41.5 | –39.8 |
| 14 | NPA014089 | –8.0 | –39.8 | –39.7 |
| 15 | NPA007540 | –7.8 | –42.7 | –39.7 |
| 16 | NPA012852 | –9.0 | –41.2 | –39.5 |
| 17 | NPA018545 | –10.4 | –40.3 | –39.4 |
| 18 | NPA002806 | –9.4 | –42.6 | –38.4 |
| 19 | NPA019342 | –8.4 | –38.5 | –37.9 |

^a^ Data ranked based on the MM-GBSA binding energy over the 25 ns MDS.

**Table S4.** Estimated covalent docking scores and MM-GBSA binding energies (in kcal.mol^−1^) over 5, 25, and 100 ns MDS of the top 11 NPAtlas compounds and PBTZ169 against DprE1^a^.

| **No.** | **NPAtlas Code** | **Covalent Docking Score (kcal.mol^−1^)** | **MM-GBSA Binding Energy (kcal.mol^−1^)** | | |
| --- | --- | --- | --- | --- | --- |
|  |  |  | **5 ns** | **25 ns** | **100 ns** |
|  | PBTZ169 (Macozinone) | –7.8 | –37.9 | –42.9 | –49.8 |
| 1 | NPA011203 | –15.8 | –75.7 | –73.3 | –73.6 |
| 2 | NPA013234 | –15.3 | –68.6 | –63.3 | –62.1 |
| 3 | NPA016048 | –15.1 | –62.2 | –62.0 | –61.8 |
| 4 | NPA001712 | –14.4 | –49.4 | –52.4 | –56.0 |
| 5 | NPA012944 | –14.7 | –56.1 | –52.8 | –53.2 |
| 6 | NPA002823 | –13.9 | –56.9 | –52.7 | –51.1 |
| 7 | NPA010851 | –13.8 | –46.2 | –47.8 | –48.0 |
| 8 | NPA007530 | –8.1 | –40.4 | –43.0 | –43.3 |
| 9 | NPA029875 | –10.4 | –47.3 | –43.8 | –42.4 |
| 10 | NPA030190 | –8.2 | –43.1 | –45.1 | –42.0 |
| 11 | NPA014008 | –10.1 | –57.1 | –46.1 | –40.5 |

^a^ Data ranked according to the MM-GBSA binding energy over the 100 ns MDS.

**Table S5.** Estimated number of the rotatable bond (RB), synthetic accessibility (SA) score, and bioavailability (BA) score of the identified NPAtlas compounds and PBZ169.

| **NPAtlas Code** | **RB** | **SA score** | **BA score** |
| --- | --- | --- | --- |
| PBTZ169 (Macozinone) | 5 | 3.82 | 0.55 |
| NPA011203 | 5 | 5.10 | 0.55 |
| NPA013234 | 27 | 4.10 | 0.17 |
| NPA016048 | 24 | 6.38 | 0.11 |
| NPA012944 | 5 | 5.03 | 0.55 |
| NPA001712 | 5 | 4.81 | 0.17 |
| NPA002823 | 10 | 9.88 | 0.17 |
